# Supplementary material for: Activity of Defined Mushroom Body Output Neurons Underlies Learned Olfactory Behavior in Drosophila
Source: Neuron. 2015 Apr 22;86(2):417–27. doi: 10.1016/j.neuron.2015.03.025 (PMC4416108; doi:10.1016/j.neuron.2015.03.025)
Supplement: Document S1. Figures S1–S8 [file mmc1.pdf]

Neuron

Supplemental Information

Activity of Defined Mushroom Body Output Neurons Underlies Learned Olfactory Behavior in *Drosophila*

David Owald, Johannes Felsenberg, Clifford B. Talbot, Gaurav Das, Emmanuel Perisse, Wolf Huetteroth, and Scott Waddell

Inventory for Supplemental Information

Figure S1. (A) Additional data for GRASP experiment in Figure 1F. Whole brain expression pattern of R48B04-LexA driver line used for GRASP in Figure 1F. (B) Similar GRASP signal observed with independent dopaminergic and output neuron driver lines; R58E02-LexA and R66C08-GAL4.

Figure S2. Permissive temperature controls for experiments in Figure 2.

Figure S3. Odor-evoked GCaMP responses in the gamma lobe output to complement responses shown in Figure 3A-D.

Figure S4. Physiological responses to additional odors and behavioral memory tests using these odors. (A) relates to Figure 3A-D. (B) relates to Figure 2A and Figure 3.

Figure S5. Permissive temperature controls for experiments in Figure 3E-F.

Figure S6. Normalized raw data traces for Figure 3I-L.

Figure S7. Example data analysis for odor response curves shown in Figure 3I-J.

Figure S8. Permissive temperature controls for experiments in Figure 4.

Movie S1. Projection view of innervation relating to Figure 1A.

Movie S2. Projection view of innervation relating to Figure 1B.

Movie S3. Projection view of innervation relating to Figure 1C.

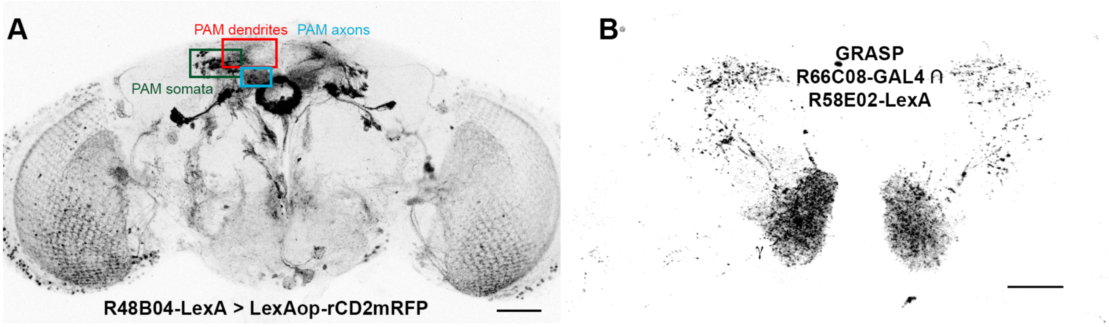

Figure S1. (A) Projection view of a brain from a R48B04-LexA; LexAop-rCD2-mRFP fly. Note that the ellipsoid body expression is in a different plane to that of PAM cells. Scale bar is 50 μm. (B) GRASP reveals sites of putative contact between M6 neurons and the rewarding dopaminergic neurons. Dopaminergic neurons expressed one half of the split GFP with R58E02-LexA and the complementary fragment was driven in M6/MBON-γ5β´2a neurons by R66C08-GAL4. Also compare Figure 1F for GRASP with dopaminergic neurons labeled with R48B04-LexA and M6/MBON-γ5β´2a and M4β´/MBON-β´2mp with VT1211-GAL4. Scale bar is 20 μm.

	immediate appetitive	24h appetitive	immediate aversive
R66C08-GAL4	n = 10<br>0.34 ± 0.04<br>$p > 0.05$	n = 7<br>0.40 ± 0.04<br>$p > 0.05$	n = 15<br>-0.55 ± 0.04<br>$p > 0.05$ ♯
UAS-*shi*^{ts1}	n = 11<br>0.24 ± 0.06<br>$p > 0.05$	n = 5<br>0.47 ± 0.04 ‡<br>$p > 0.05$	n = 6<br>-0.34 ± 0.04<br>$p > 0.05$ ♯
R66C08 > *shi*^{ts1}	n = 10<br>0.28 ± 0.05<br>$p > 0.05$	n = 4<br>0.50 ± 0.04<br>$p > 0.05$	n = 12<br>-0.45 ± 0.04<br>$p > 0.05$
VT1211-GAL4	n = 9<br>0.21 ± 0.02<br>$p > 0.05$	n = 5<br>0.32 ± 0.04<br>$p > 0.05$	n = 10<br>-0.36 ± 0.04<br>$p > 0.05$
UAS-*shi*^{ts1}	n = 9<br>0.30 ± 0.04<br>$p > 0.05$	n = 5<br>0.47 ± 0.04 ‡<br>$p > 0.05$	n = 10<br>-0.31 ± 0.07<br>$p > 0.05$
VT1211 > *shi*^{ts1}	n = 9<br>0.21 ± 0.03<br>$p > 0.05$	n = 7<br>0.29 ± 0.07<br>$p > 0.05$	n = 11<br>-0.29 ± 0.05<br>$p > 0.05$
R21D02-GAL4	n = 8<br>0.30 ± 0.06<br>$p > 0.05$	n = 6<br>0.16 ± 0.04<br>$p > 0.05$	n = 4<br>-0.23 ± 0.08<br>$p > 0.05$
UAS-*shi*^{ts1}	n = 6<br>0.27 ± 0.01<br>$p > 0.05$	n = 7<br>0.20 ± 0.03<br>$p > 0.05$	n = 4<br>-0.17 ± 0.04<br>$p > 0.05$
R21D02 > *shi*^{ts1}	n = 7<br>0.25 ± 0.07<br>$p > 0.05$	n = 7<br>0.10 ± 0.03<br>$p > 0.05$	n = 4<br>-0.17 ± 0.04<br>$p > 0.05$

Figure S2. Permissive temperature control experiments for the restrictive temperature memory experiments shown in Figure 2. ‡ indicates data sets acquired in parallel. # indicates the R66C08-GAL4 and UAS-*shi*^{ts1} flies were statistically different. Data shown are mean ± SEM.

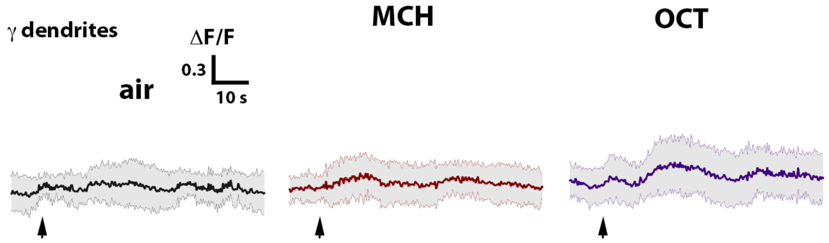

Figure S3. Time courses of odor-evoked GCaMP responses (ΔF/F) collected at the level of the M6/MBON-γ5β´2a neuron dendrites in the γ lobe. Traces represent the mean odor responses (solid line) and standard deviation (grey shading), n = 19. Arrows indicate onset of odor presentation. Also compare Figure 3C for M4β´/MBON-β´2mp responses.

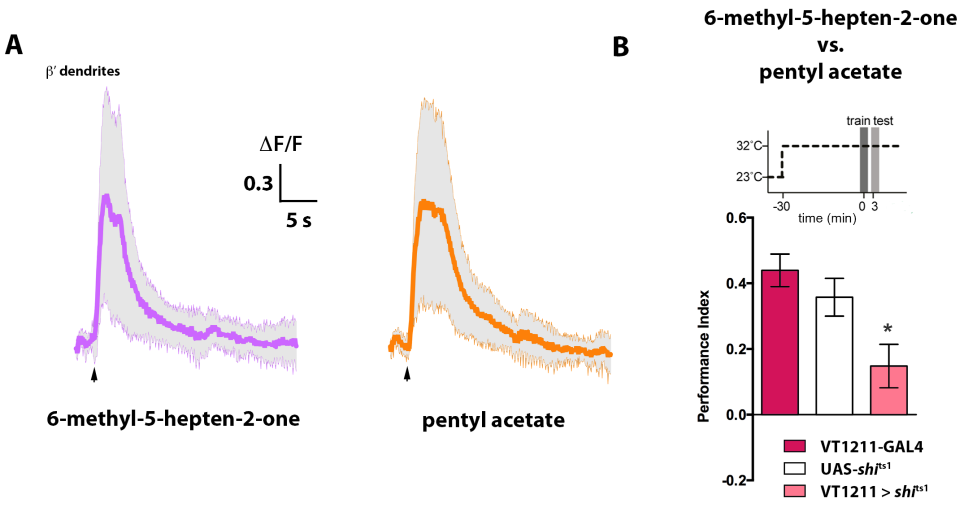

Figure S4. (A) Time courses of odor-evoked GCaMP responses (ΔF/F) collected from M4β′/MBON-β′2mp dendrites to a 5 s 6-methyl-5-hepten-2-one and pentyl acetate exposure. Traces represent the mean odor responses (solid line) and standard deviation (grey shading), n = 16. Arrows indicate onset of odor presentation. (B) Blocking M4β′/MBON-β′2mp and M6/MBON-γ5β′2a (VT1211-Gal4) neurons with UAS-*shibire*^{ts1} significantly impairs 3 min appetitive memory performance when trained with 6-methyl-5-hepten-2-one and pentyl acetate ($n \geq 8$, $P < 0.05$). Data shown are mean ± SEM.

	2 h appetitive	2 h aversive
VT1211-GAL4	n = 5<br>0.14 ± 0.07<br>p > 0.05	n = 12<br>-0.18 ± 0.05<br>p > 0.05
UAS-*shi*^{ts1}	n = 6<br>0.21 ± 0.05<br>p > 0.05	n = 12<br>-0.16 ± 0.03<br>p > 0.05
VT1211 > *shi*^{ts1}	n = 5<br>0.16 ± 0.07<br>p > 0.05	n = 13<br>-0.19 ± 0.05<br>p > 0.05

Figure S5. Permissive temperature controls for the behavioral experiments shown in Figure 3. Data shown are mean ± SEM.

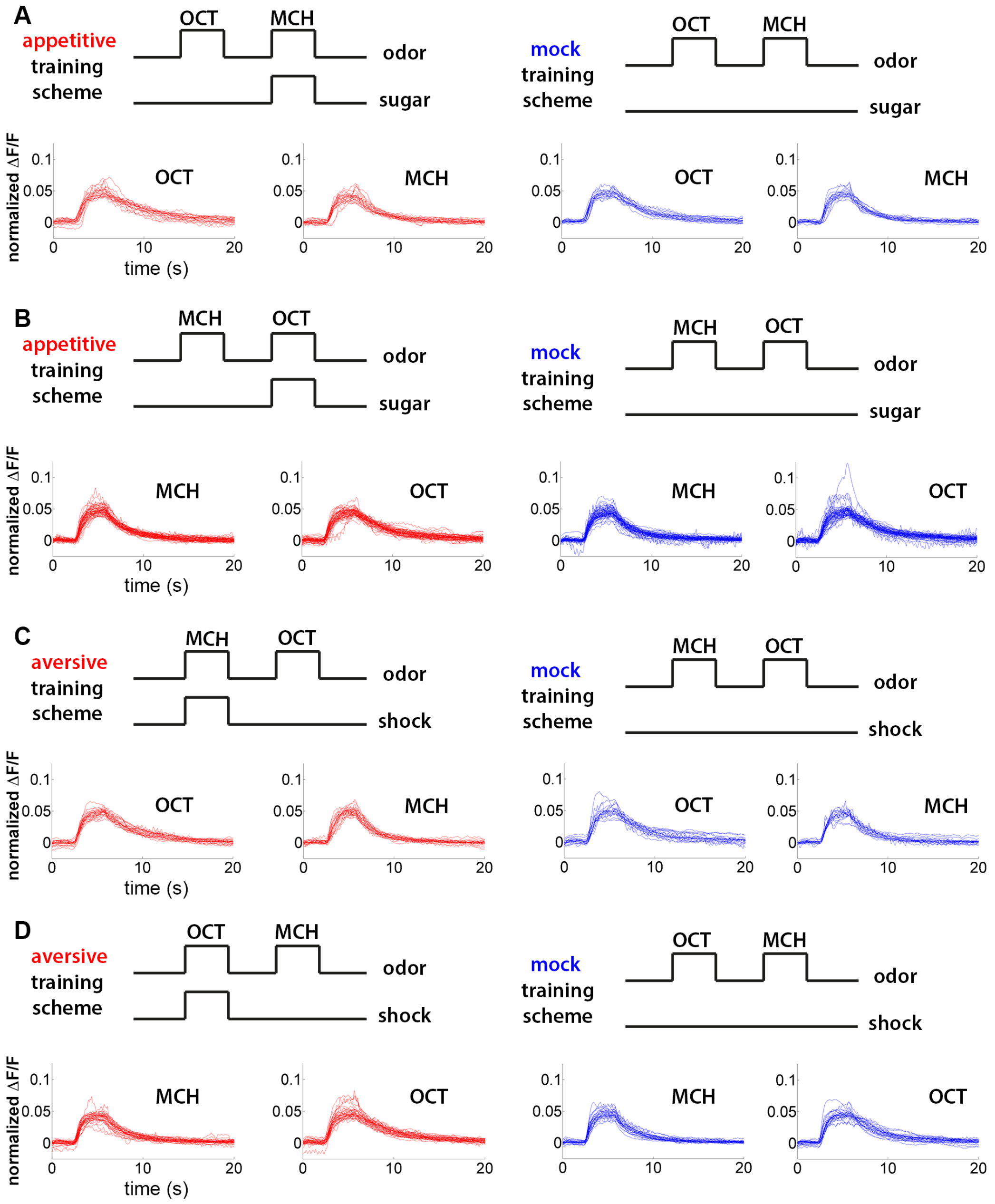

Figure S6. Response traces for each 'n' for data shown in Figure 3I-L. Individual traces are averages of two odor presentations as described in the methods. Conditioning protocols are illustrated in upper panels of A to D and normalized response traces (see methods, equation (i)) are shown in lower panels. (A) appetitive, MCH is CS+ (B) appetitive, OCT is CS+ (C) aversive, MCH is CS+ (D) aversive, OCT is CS+.

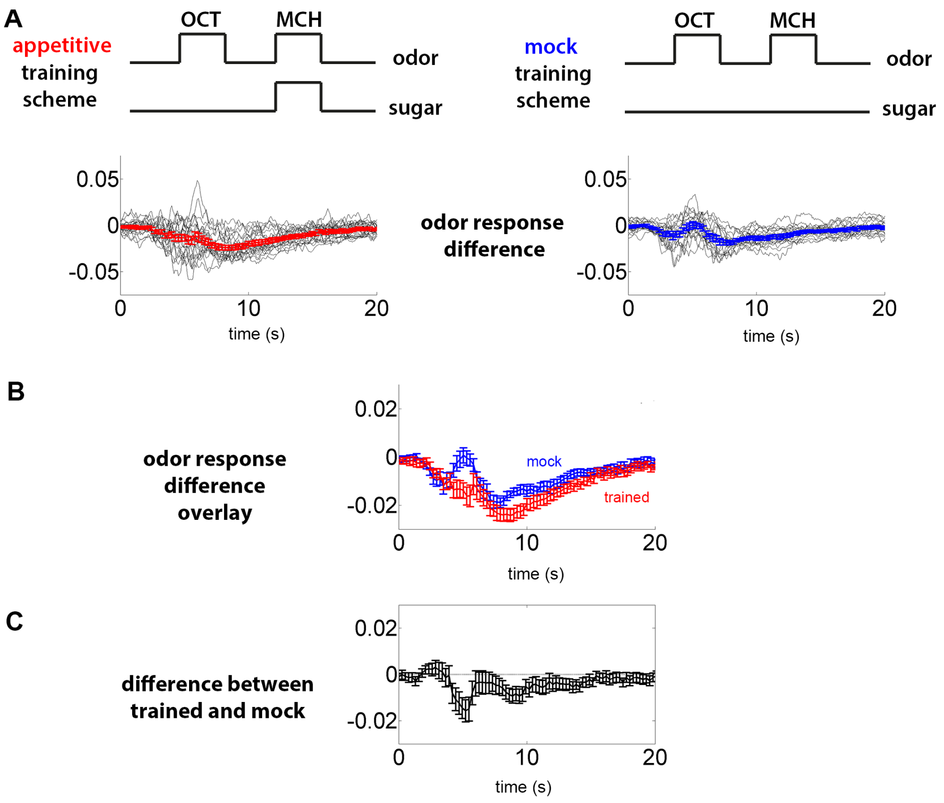

Figure S7. Example of data analysis for odor response curves shown in Figure 3I and 3J. (A) Above: Schematic of the conditioning protocol. Below: The odor response difference curves (see methods). OCT traces are subtracted from the MCH traces per individual trained fly (grey lines). The red (trained) and blue (mock trained) lines are both mean ± SEM. (B) Overlay of the averaged odor response difference curves for the trained (red line ± SEM) and mock trained (blue line ± SEM) flies. (C) The mean odor response difference curves are subtracted in order to compare the averaged responses from trained flies to those from mock trained flies, mean ± SEM. Methods contain further detail.

	MCH	OCT
R21D02-Gal4	n = 13<br>-0.33 ± 0.07<br>p > 0.05	n = 10<br>-0.16 ± 0.07<br>p > 0.05
VT1211-Gal4	n = 13<br>-0.33 ± 0.05<br>p > 0.05	n = 7<br>-0.32 ± 0.05<br>p > 0.05
R66C08-Gal4	see below	n = 6<br>-0.48 ± 0.08<br>p > 0.05
UAS-*shi*^{ts1}	n = 15<br>-0.62 ± 0.03 #<br>p < 0.05	n = 10<br>-0.32 ± 0.07<br>p > 0.05
R21D02 > *shi*^{ts1}	n = 10<br>-0.21 ± 0.06<br>p > 0.05	n = 7<br>-0.33 ± 0.07<br>p > 0.05
VT1211 > *shi*^{ts1}	n = 15<br>-0.35 ± 0.04<br>p > 0.05	n = 6<br>-0.36 ± 0.13<br>p > 0.05
R66C08 > *shi*^{ts1}	see below	n = 6<br>-0.45 ± 0.09<br>p > 0.05
R66C08-Gal4	n = 6<br>-0.38 ± 0.03<br>p < 0.05	see above
UAS-*shi*^{ts1}	n = 6<br>-0.23 ± 0.09<br>p < 0.05	see above
R66C08 > *shi*^{ts1}	n = 6<br>-0.30 ± 0.09<br>p > 0.05	see above

Figure S8. Permissive temperature control experiments for the restrictive naïve odor choice experiments shown in Figure 4. # indicates the UAS-*shi*^{ts1} flies showed statistical difference to relevant groups. Data shown are mean ± SEM.
