# Supplementary figures and images for: Activity of Defined Mushroom Body Output Neurons Underlies Learned Olfactory Behavior in Drosophila
Source: Neuron. 2015 Apr 22;86(2):417–27. doi: 10.1016/j.neuron.2015.03.025 (PMC4416108; doi:10.1016/j.neuron.2015.03.025)

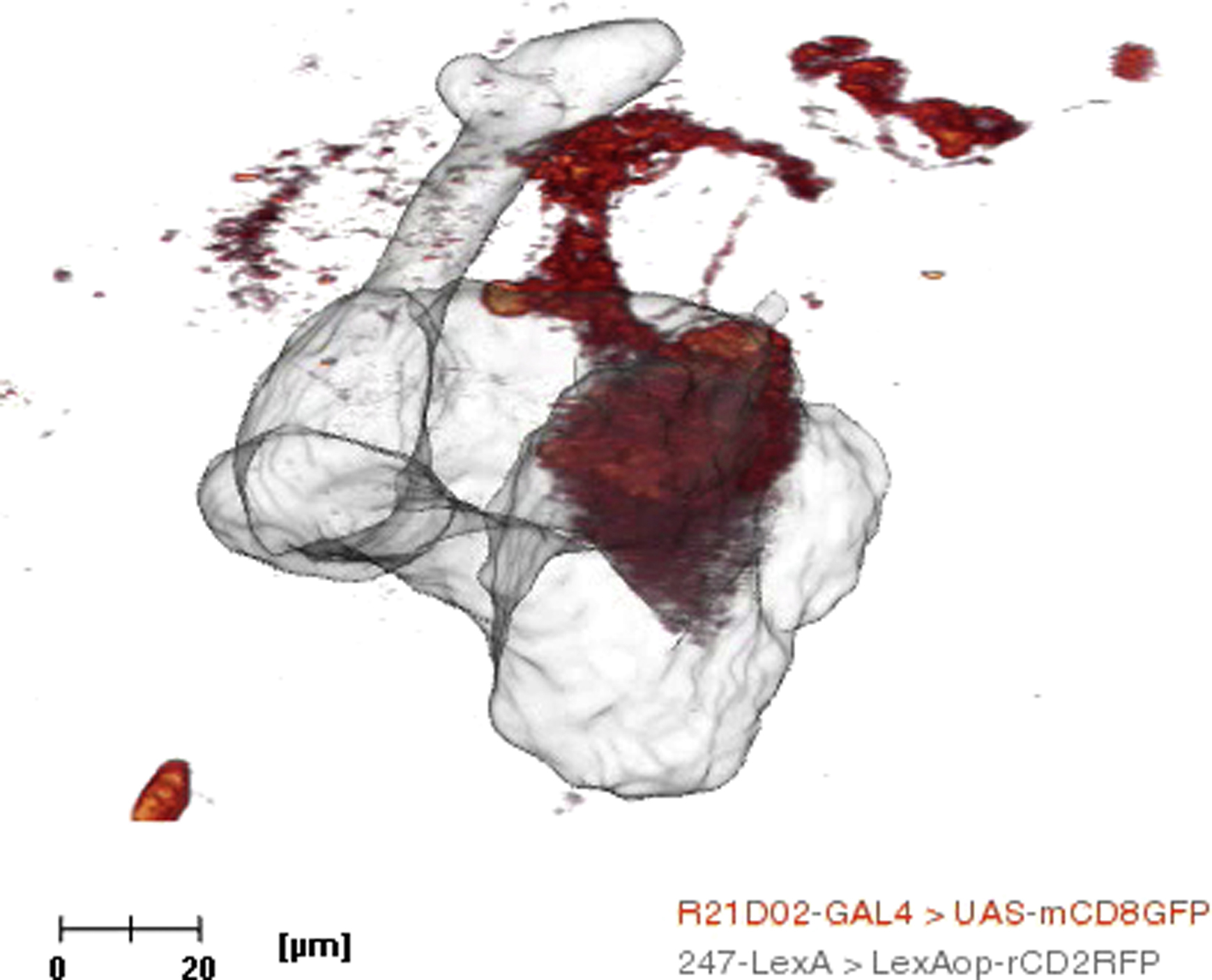

Supplement: Movie S1. Projection View of Innervation Relating to Figure 1A — Projection view of the innervation of M4β/MBON-β2β′2a, M4β′/MBON-β′2mp and M6/MBON-γ5β′2a neurons in the mushroom body β, β′, and γ lobe tips from a R21D02-GAL4 brain. [file mmc2.jpg]

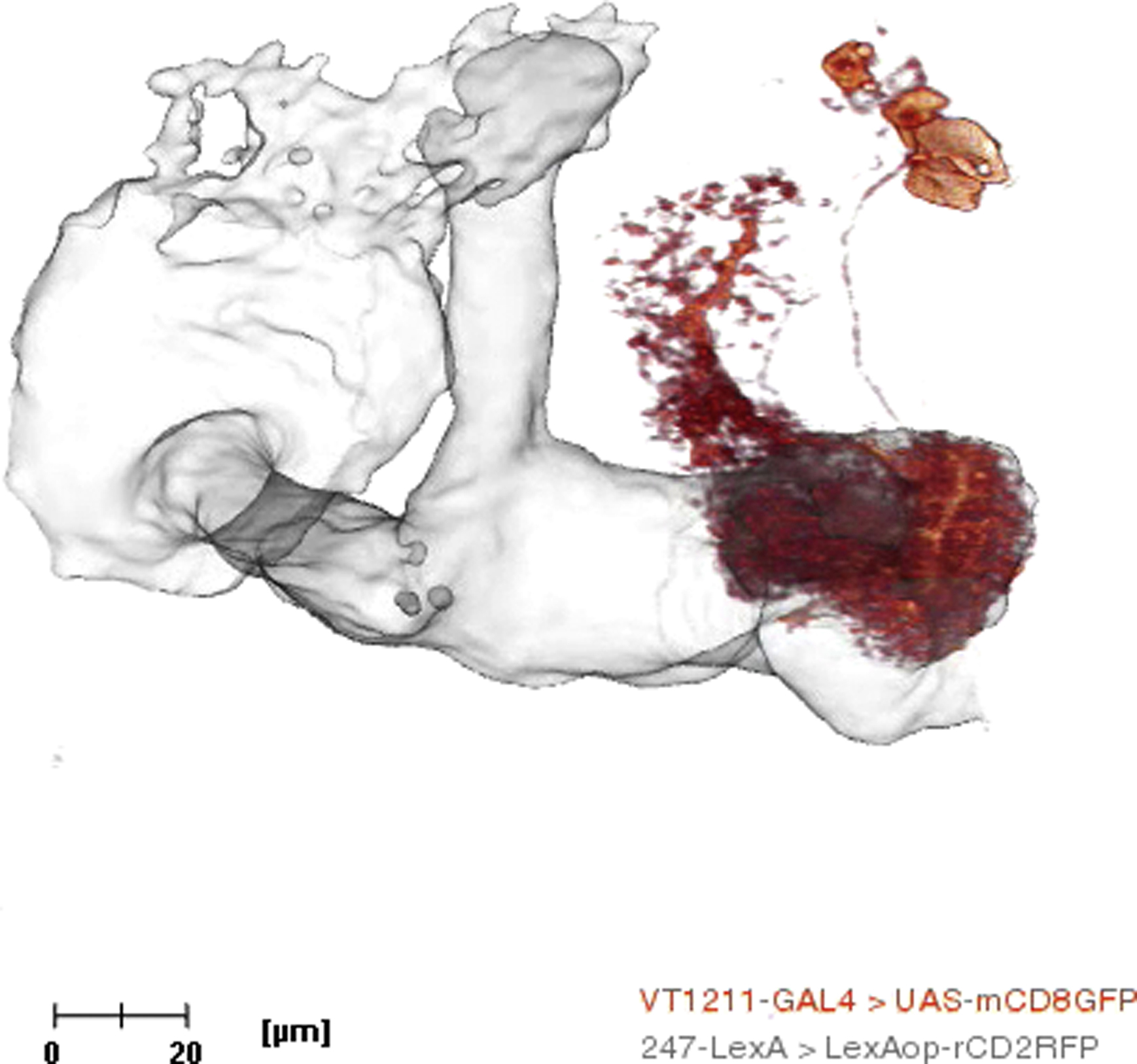

Supplement: Movie S2. Projection View of Innervation Relating to Figure 1B — Projection view of the innervation of M4β′/MBON-β′2mp and M6/MBON-γ5β′2a neurons in the mushroom body β′ and γ lobe tips from a VT1211-GAL4 brain. [file mmc3.jpg]

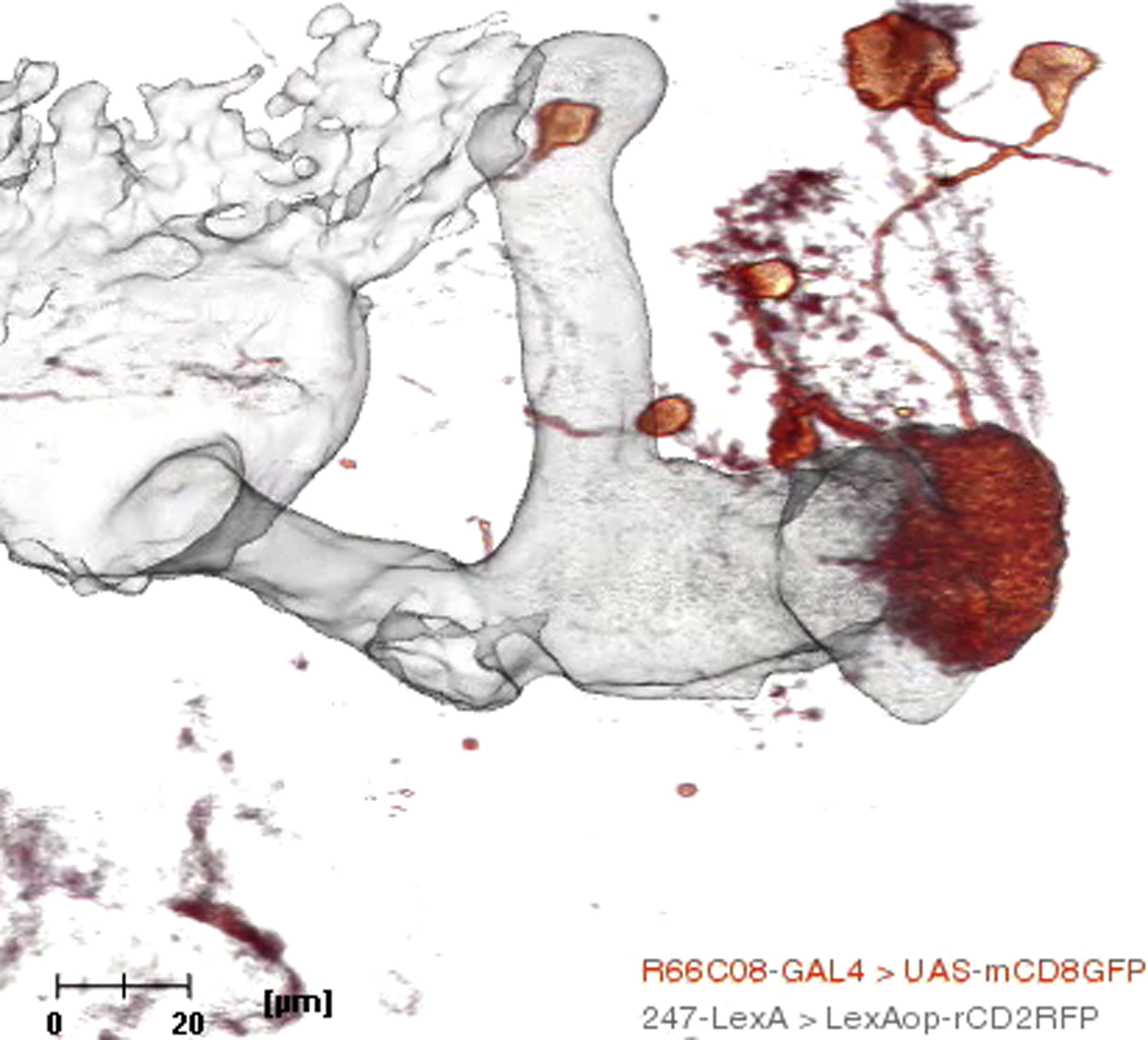

Supplement: Movie S3. Projection View of Innervation Relating to Figure 1C — Projection view of the innervation of M6/MBON-γ5β′2a neurons in the mushroom body γ lobe tip from a R66C08-GAL4 brain. [file mmc4.jpg]
