# Supplementary material for: Activity of Defined Mushroom Body Output Neurons Underlies Learned Olfactory Behavior in Drosophila
Source: Neuron. 2015 Apr 22;86(2):417–27. doi: 10.1016/j.neuron.2015.03.025 (PMC4416108; doi:10.1016/j.neuron.2015.03.025)
Supplement: Document S2. Article plus Supplemental Information [file mmc5.pdf]

Neuron

Article

Activity of Defined Mushroom Body Output Neurons Underlies Learned Olfactory Behavior in *Drosophila*

Highlights

- Glutamatergic mushroom body output neurons are required for memory expression
- Training bidirectionally alters relative odor drive to output neurons
- Blocking glutamatergic mushroom body output neurons mimics appetitive conditioning
- Optogenetic activation drives avoidance behavior

Authors

David Owald, Johannes Felsenberg, ..., Wolf Huetteroth, Scott Waddell

Correspondence

scott.waddell@cncb.ox.ac.uk

In Brief

Fruit fly olfactory memory involves mushroom body plasticity. Owald et al. identified glutamatergic mushroom body output neurons that are critical for memory expression. Conditioning bidirectionally alters odor drive to these outputs. Blocking them mimics appetitive conditioning, whereas activation induces avoidance behavior.

Owald et al., 2015, Neuron *86*, 417–427
April 22, 2015 ©2015 The Authors
http://dx.doi.org/10.1016/j.neuron.2015.03.025

Neuron
Article

Activity of Defined Mushroom Body Output Neurons Underlies Learned Olfactory Behavior in *Drosophila*

David Owald,[1] Johannes Felsenberg,[1] Clifford B. Talbot,[1] Gaurav Das,[1] Emmanuel Perisse,[1] Wolf Huetteroth,[1] and Scott Waddell[1,*]
[1]Centre for Neural Circuits and Behaviour, The University of Oxford, Tinsley Building, Mansfield Road, Oxford OX1 3SR, UK
*Correspondence: scott.waddell@cncb.ox.ac.uk
http://dx.doi.org/10.1016/j.neuron.2015.03.025
This is an open access article under the CC BY license (http://creativecommons.org/licenses/by/4.0/).

SUMMARY

During olfactory learning in fruit flies, dopaminergic neurons assign value to odor representations in the mushroom body Kenyon cells. Here we identify a class of downstream glutamatergic mushroom body output neurons (MBONs) called M4/6, or MBON-β2β′2a, MBON-β′2mp, and MBON-γ5β′2a, whose dendritic fields overlap with dopaminergic neuron projections in the tips of the β, β′, and γ lobes. This anatomy and their odor tuning suggests that M4/6 neurons pool odor-driven Kenyon cell synaptic outputs. Like that of mushroom body neurons, M4/6 output is required for expression of appetitive and aversive memory performance. Moreover, appetitive and aversive olfactory conditioning bidirectionally alters the relative odor-drive of M4β′ neurons (MBON-β′2mp). Direct block of M4/6 neurons in naive flies mimics appetitive conditioning, being sufficient to convert odor-driven avoidance into approach, while optogenetically activating these neurons induces avoidance behavior. We therefore propose that drive to the M4/6 neurons reflects odor-directed behavioral choice.

INTRODUCTION

Learning permits animals to convert innate reflexive stimulus-driven behavioral responses into meaningful stimulus-guided actions. Understanding how such sensory-motor transformations are implemented and altered in the nervous system is a subject of great interest.

In *Drosophila*, innate behavioral responses to odors can be redirected toward approach or avoidance by a learning session that couples odor exposure with rewarding sugar or punitive electric shock, respectively (Tempel et al., 1983; Tully and Quinn, 1985). Recently, substantial progress has been made in understanding the neural mechanisms of odorant coding and learning in the fly (Wilson, 2013; Masse et al., 2009; Perisse et al., 2013; Busto et al., 2010; Dubnau and Chiang, 2013). However, it remains unclear how peripheral odor responses are transformed into behavioral performance and how learning redirects the transformation.

Flies detect airborne odors using unique collections of olfactory sensory neurons (OSNs) housed in their antennae and maxillary palps (de Bruyne et al., 1999, 2001). The tuning of each OSN type is determined by the expression of a single odorant receptor gene (Dobritsa et al., 2003; Hallem and Carlson, 2004, 2006; Vosshall, 2000). Axons from OSNs expressing the same receptor converge onto the same glomerulus in each antennal lobe (Vosshall, 2000; Gao et al., 2000; Couto et al., 2005; Fishilevich and Vosshall, 2005), where their activity is relayed to excitatory and inhibitory projection neurons (Olsen et al., 2010; Kazama and Wilson, 2008; Parnas et al., 2013; Liang et al., 2013). Excitatory projection neurons deliver odor information to the calyces of the mushroom bodies (MBs) and to neurons in the lateral horn (LH), whereas inhibitory PN activity is exclusively relayed to the LH (Jefferis et al., 2001; Wong et al., 2002; Fişek and Wilson, 2014; Wang et al., 2014). The LH is largely believed to be responsible for driving innate behavioral responses to odors, since blocking all mushroom body neuron output has little consequence on these behaviors (Heimbeck et al., 2001; Parnas et al., 2013). In contrast, disrupting the mushroom body has long been known to impair learned responses (Heisenberg et al., 1985; Dubnau et al., 2001; McGuire et al., 2001; Schwaerzel et al., 2002), consistent with the MB being critical for odor memory (Heisenberg, 2003).

Each MB is comprised of 2,000 intrinsic Kenyon cells (KCs), and an individual odor is represented as activity in a sparse subset of these cells (Wang et al., 2004; Honegger et al., 2011). Value can be assigned to these odor representations during learning by the action of reinforcing dopaminergic neurons whose presynaptic terminals are confined to discrete zones along the lobes of the MB (Schwaerzel et al., 2003; Riemensperger et al., 2005; Claridge-Chang et al., 2009; Aso et al., 2012; Mao and Davis, 2009; Liu et al., 2012; Burke et al., 2012; Waddell, 2013). This anatomy and a requirement for dopamine receptor in MB neurons (Kim et al., 2007; Qin et al., 2012) is consistent with a model that olfactory memories are represented in the presynaptic output synapses from mushroom body KCs onto relevant downstream neurons (Heisenberg, 2003).

Anatomical work suggests that fewer than 40 output neurons collect synaptic outputs from the 2,000 KCs (Tanaka et al., 2008; Aso et al., 2014). This substantial convergence indicates that information may be lost, and raises the question of what information is represented as changes in synaptic efficacy from KCs to downstream output neurons. Prior work suggests that the MB is involved in motor gating (Huber, 1967; Martin et al.,

1998) and that an element of memory valence is differentially coded between subclasses of the αβ KCs (Perisse et al., 2013). How such information is represented in the connections between KCs and particular downstream neurons is currently unclear.

Physiological changes after training have been reported in two sets of memory-relevant cholinergic output neurons that have dendritic fields within the vertical lobes of the mushroom body (Séjourné et al., 2011; Pai et al., 2013; Plaçais et al., 2013). However, the behavioral consequence of synaptic modification at these sites is unclear. Here we identify a small set of glutamatergic output neurons whose dendrites lie within the tip regions of the horizontal mushroom body lobes and in close spatial proximity to presynaptic terminals of reinforcing dopaminergic neurons (Burke et al., 2012; Liu et al., 2012). Blocking these output neurons impairs conditioned odor approach and avoidance. Strikingly, the activation of these output neurons by the conditioned odor is depressed by reward learning and potentiated by aversive learning. Moreover, directly inhibiting these neurons in naive flies converts odor avoidance into attraction, whereas flies are repelled by their activation. Our data therefore suggest that a critical element of learning-induced plasticity within the MB manifests as a bidirectional change in the relative odor drive to specific types of MB output neurons.

RESULTS

GAL4 Control of Glutamatergic M4/6 MBONs

Identified dopaminergic neurons in the PAM (protocerebral anterior medial) cluster in the *Drosophila* brain convey rewarding reinforcement (Burke et al., 2012; Liu et al., 2012). Blocking the output from a subset of these that are labeled by the 0104-GAL4 driver impairs short-term sweetness-reinforced and longer-term nutrient-reinforced sugar memory (Burke et al., 2012). Furthermore, pairing thermogenetic activation of these neurons with odor presentation formed appetitive odor memories (Burke et al., 2012). The presynaptic terminals from 0104-labeled dopaminergic neurons densely innervate the β′ and γ lobe tips of the horizontal mushroom body lobes, which suggests that appetitive olfactory memories may be represented as changes in the efficacy of synaptic outputs in these regions from the odor-activated KCs onto as-yet-unidentified downstream neurons.

By visually screening available GAL4 collections (Jenett et al., 2012; Bidaye et al., 2014), we identified three fly lines that labeled candidate postsynaptic neurons with arbors in the tip regions, β_2, β'_2, and γ_5, of the horizontal mushroom body lobes (Figure 1). Neurons innervating β'_2 and γ_5 have been described as MB-M4 and MB-M6 (Tanaka et al., 2008). We therefore named the cells that predominantly innervate either the tip of the β, β′, or γ lobe as M4β, M4β′, and M6, respectively. A very recent study has renamed these neurons as MBON-β2β′2a (M4β), MBON-β′2mp (M4β′), and MBON-γ5β′2a (M6) (Aso et al., 2014). We use both names here for clarity. R21D02-GAL4 expresses in all M4β/MBON-β2β′2a, M4β′/MBON-β′2mp, and M6/MBON-γ5β′2a neurons per hemisphere (Figure 1A, Movie S1). VT1211-GAL4 expresses in M4β′/MBON-β′2mp and M6/MBON-γ5β′2a, but not in the β tip projecting M4β/MBON-β2β′2a (Figure 1B, Movie S2). Lastly, R66C08-GAL4 only expresses in the M6/MBON-γ5β′2a neurons that mostly innervate the γ lobe tip and the anterior zone of β'_2 (Figure 1C, Movie S3). We determined the polarity of the M4/6 neurons using expression of established neural compartment marker proteins. The dendritic marker DenMark (Nicolaï et al., 2010) localized exclusively to the horizontal MB lobe tips, while the presynaptic active zone protein Syd-1 (Owald et al., 2010) localized to the processes of the M4/6 neurons that lie outside of the MB in the superior medial protocerebrum (SMP) and the crepine region (Ito et al., 2014) (Figure 1D). This polarity suggests that the dendritic field of the M4/6 neurons lies within the MB lobes and is consistent with a role as potential output neurons that pool KC synaptic weights. The genomic fragment used to create the VT1211-GAL4 line (Bidaye et al., 2014) comes from a region that is proximal to the gene for the vesicular glutamate transporter (DVGlut) (Daniels et al., 2008; Mahr and Aberle, 2006). We immunostained the fly brain with an anti-DVGlut antibody (Mahr and Aberle, 2006) to determine whether the M4/6 neurons might be glutamatergic. DVGlut labeling perfectly overlapped with the GFP-marked presynaptic field of the M4/6 neurons (Figure 1E). This is most evident at higher resolution where, in addition, individual M4/6 presynaptic boutons can be seen to be large and spherical (Figure 1E, inserts). We also used GRASP (Feinberg et al., 2008; Gordon and Scott, 2009) to test whether the processes of the M4/6 neurons are close to those of the dopaminergic PAM neurons (Figures 1F and S1B). This analysis revealed strong GFP fluorescence at two locations: the tips of the horizontal MB lobes, where the M4/6 dendrites and dopaminergic presynapses reside, and in the SMP between M4/6 presynaptic terminals and the dendrites of dopaminergic neuron. Although GRASP is most reliably a proximity marker, it is notable that the GRASP visible in the SMP appears to preferentially label terminals of M4/6 neurons rather than the non-synaptic neurites, suggesting that the points of contact may be genuinely synaptic.

M4/6 Neurons Are Required for Appetitive and Aversive Memory Expression

We tested whether output from M4/6 neurons was required for behavioral expression of memory performance by using the R21D02, VT1211, and R66C08 GAL4 drivers to express the dominant temperature-sensitive UAS-*shibire*^{ts1} (*shi*^{ts1}) transgene (Kitamoto, 2001). In each experiment we compared the performance of flies with M4/6 neural blockade to control flies carrying only the GAL4 or UAS-*shi*^{ts1} transgene. We first tested immediate memory performance following sucrose-reinforced appetitive conditioning (Tempel et al., 1983; Krashes and Waddell, 2008). All flies were trained and tested for 3 min memory at the restrictive temperature of 32°C. Blocking the M4/6 neurons caused an impairment in memory performance. R21D02;*shi*^{ts1}, VT1211;*shi*^{ts1} and R66C08;*shi*^{ts1} flies displayed performance that was statistically different to that of *shi*^{ts1} and their respective GAL4 control flies (Figures 2A1, 2B1, and 2C1). We also restricted the blockade of M4/6 neurons to the time of memory retrieval by training flies at the permissive 23°C and raising the temperature to 32°C 30 min before and during testing 24 hr appetitive memory. These analyses again uncovered a significant defect in flies with impaired M4/6 neurons, demonstrating a clear requirement for M4/6 neural output for the

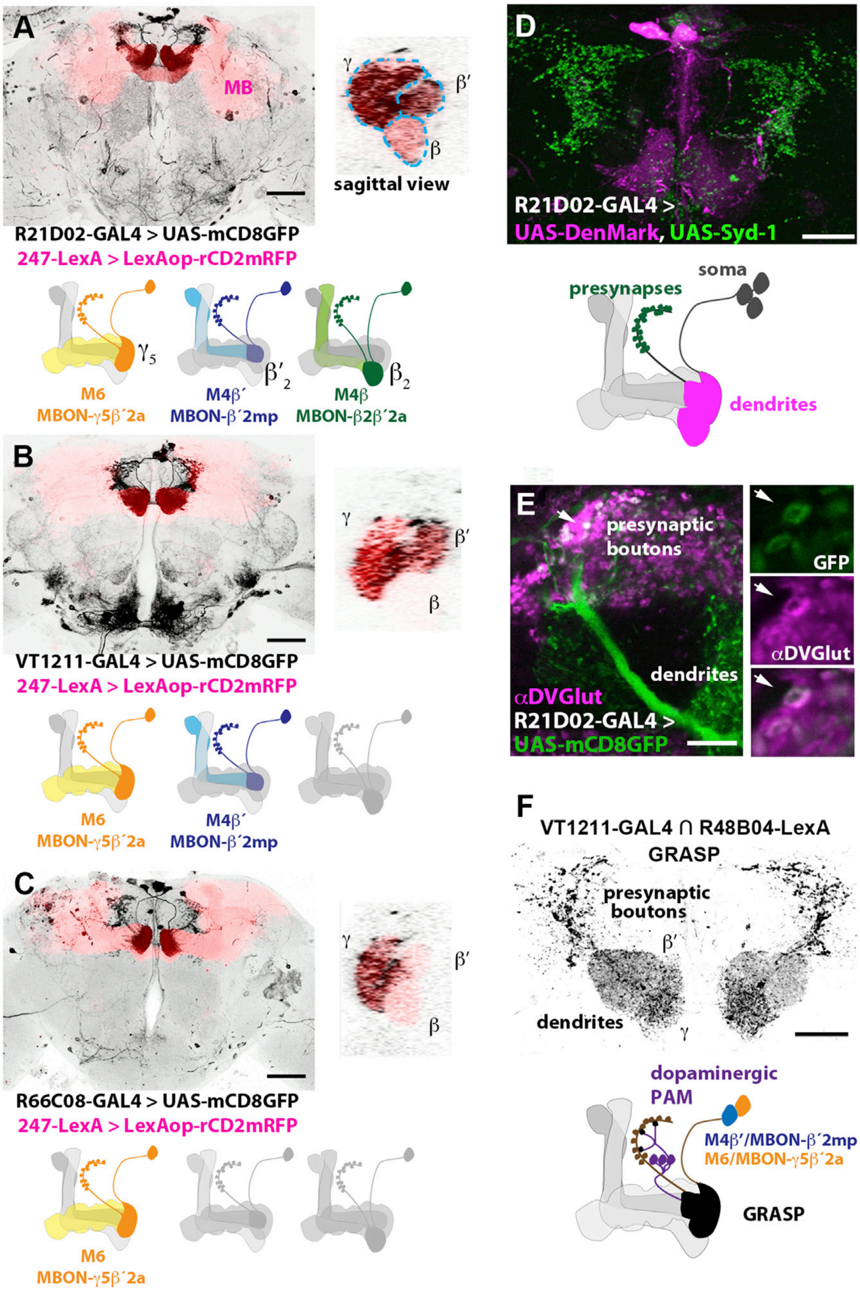

Figure 1. Three Pairs of Glutamatergic M4β/MBON-β2β′2a, M4β′/MBON-β′2mp, and M6/MBON-γ5β′2a Output Neurons Innervate the Tips of the Horizontal Mushroom Body Lobes

(A–C) The M4β/MBON-β2β′2a, M4β′/MBON-β′2mp, and M6/MBON-γ5β′2a neurons predominantly innervate either the tips of the β, β′, or γ lobes of the mushroom bodies (MB). (A) R21D02-GAL4 expresses in the M4β/MBON-β2β′2a, M4β′/MBON-β′2mp, and M6/MBON-γ5β′2a neurons that predominantly innervate the β, β′, and γ lobe, respectively. (B) VT1211-GAL4 labels the M4β′/MBON-β′2mp and M6/MBON-γ5β′2a that innervate the β′ and γ lobes. (C) R66C08-GAL4 only expresses in the two M6/MBON-γ5β′2a neurons that predominantly innervate the γ lobes but also have a projection into the anterior zone of the β′ tip. (A–C) Scale bar is 50 μm. Right panels provide magnified sagittal views through the tips of the horizontal MB lobes and illustrate the respective innervation of M4/6 neurons in the β, β′, and γ lobes (indicated by dashed lines in A). Cartoons summarize the neurons covered by each GAL4 driver. Movies S1, S2, and S3 show projection view examples of each GAL4 line.

(D) Expression of neuronal compartment markers reveals that the M4β/MBON-β2β′2a, M4β′/MBON-β′2mp, and M6/MBON-γ5β′2a neurons likely receive input from MB neurons through their DenMark-labeled dendritic region that lies within the MB lobe tips. Additionally, their Syd-1-labeled presynaptic output region is concentrated in the superior median protocerebrum (SMP) and the crepine region. Scale bar is 25 μm. Below: schematic of the polarity of M4/6 neurons.

(E) Presynaptic boutons of the M4β/MBON-β2β′2a, M4β′/MBON-β′2mp, and M6/MBON-γ5β′2a neurons (green label, white arrows) co-stain with antibody to the *Drosophila* vesicular glutamate transporter (DVGlut, magenta). Scale bar is 10 μm.

(F) GFP reconstitution across synaptic partners (GRASP) suggests that the dendrites of M4β′/MBON-β′2mp and M6/MBON-γ5β′2a neurons are in close proximity to the output regions of rewarding dopaminergic neurons in the MB lobe tips. One half of GRASP is driven by R48B04-LexA (Figure S1A) (Lin et al., 2014) and the other by VT1211-GAL4. Similar results are seen when GRASP is driven by R58E02-LexA and R66C08-GAL4 (Figure S1B). In addition, in both cases GRASP is observed between the M4β′/MBON-β′2mp and M6/MBON-γ5β′2a output synapses and the dendrites of rewarding dopaminergic neurons in the SMP. Scale bar is 20 μm.

expression of conditioned approach (Figures 2A2, 2B2, and 2C2). We similarly tested the role of M4/6 neurons in electric-shock-reinforced aversive short-term memory. Memory performance of R21D02;*shi*^{ts1}, VT1211;*shi*^{ts1} and R66C08;*shi*^{ts1} flies was again statistically different to that of *shi*^{ts1} and their respective GAL4 control flies (Figures 2A3, 2B3, and 2C3). In both the appetitive and aversive memory experiments, the observed defect appeared more pronounced when simultaneously blocking M4β′/MBON-β′2mp or M4β′/MBON-β′2mp and M4β/MBON-β2β′2a neurons with M6/MBON-γ5β′2a neurons, using VT1211 or R21D02, than blocking M6/MBON-γ5β′2a neurons alone with R66C08. Importantly, control experiments performed at permissive 23°C did not reveal significant differences between the relevant groups (Figure S2). Output from the M4β/MBON-β2β′2a, M4β′/MBON-β′2mp, and M6/MBON-γ5β′2a neurons is therefore required for the expression of appetitive and aversive memory performance and we propose that the three cell types may function together.

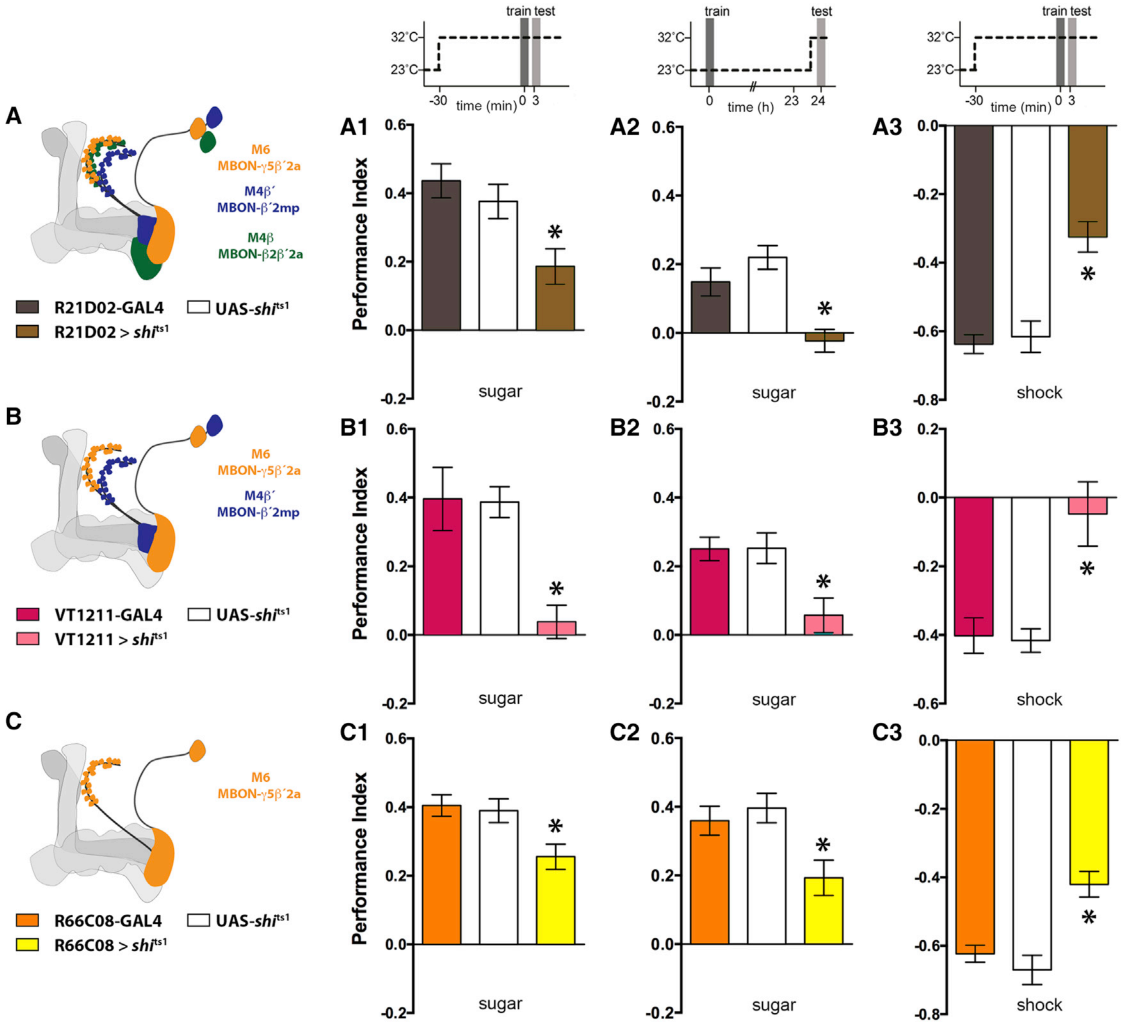

Figure 2. Blocking M4β/MBON-β2β′2a, M4β′/MBON-β′2mp, and M6/MBON-γ5β′2a Neurons Impairs the Expression of Appetitive and Aversive Memory Performance

(A–C) Schematic representations of the MBON neuron coverage in each GAL4 line used. R21D02 labels all M4β/MBON-β2β′2a, M4β′/MBON-β′2mp, and M6/MBON-γ5β′2a neurons. VT1211 labels M4β′/MBON-β′2mp and M6/MBON-γ5β′2a. R66C08 only labels the M6/MBON-γ5β′2a neurons. Blocking M4/6 neurons with UAS-*shi*^{ts1} (A1, B1, and C1) significantly impairs 3 min appetitive memory performance (A1: $n \geq 10$, $p < 0.05$; B1: $n \geq 7$, $p < 0.05$; C1: $n \geq 13$, $p < 0.05$). Blocking M4/6 neurons only during testing (A2, B2, and C2) significantly impairs 24 hr appetitive memory performance (A2: $n \geq 9$, $p < 0.05$; B2: $n \geq 16$, $p < 0.05$; C2: $n \geq 7$, $p < 0.05$). Blocking M4/6 neurons (A3, B3, C3) significantly impairs 3 min aversive memory (A3: $n \geq 10$, $p < 0.05$; B3: $n \geq 8$, $p < 0.05$; C3: $n \geq 10$, $p < 0.05$). All data are represented as the mean ± SEM. Asterisks denote $p < 0.05$; all statistics are one-way ANOVA followed by a Tukey's HSD post-hoc test.

Odors Evoke Activity in MBON Dendrites in the β′ Lobe

To further understand the role of the M4/6 neurons in shaping a behavioral response, we used VT1211-GAL4 to express GCaMP6m (Chen et al., 2013) in the M4β′/MBON-β′2mp and M6/MBON-γ5β′2a neurons and performed two-photon functional calcium imaging to monitor odor-evoked activity in living flies. We exposed flies to 5 s pulses of methylcyclohexanol (MCH) and octanol (OCT), the same odors used in training, and monitored changes in GCaMP fluorescence in the dendrites of M4β′/MBON-β′2mp and M6/MBON-γ5β′2a in the MB lobe tips (Figure 3A). The magnitude of the dendritic odor-evoked responses was smaller (and with our experimental settings below the level of noise) in MBON dendrites in the γ lobe (Figure S3) than dendrites in the β′ lobe (Figures 3B and 3C). Since the behavioral data indicated that M4β′/MBON-β′2mp and M6/MBON-γ5β′2a neurons are both required for memory performance (Figure 2B versus Figure 2C), we concentrated further analysis on the MBON dendrites in the β′ lobe. Exposing flies to MCH or OCT elicited robust calcium transients throughout the MBON dendrites in the β′ lobe (Figures 3B and 3C). Furthermore, odor-evoked calcium transients were also evident in the presynaptic boutons of the output neurons (Figure 3D), suggesting the odor-driven input to the dendrites is propagated to the release sites. The MBON dendrites in the β′ lobe also responded when flies were exposed to other odors such as 6-methyl-5-hepten-2-one and pentyl acetate (Figure S4A).

In addition, memory performance was impaired when M4β′/MBON-β′2mp and M6/MBON-γ5β′2a neurons were blocked after flies were trained with these odors (Figure S4B), suggesting that the role of the M4/6 neurons in memory retrieval is not specific to OCT and MCH.

Learning Bidirectionally Alters Relative Odor Drive to M4β′ Neurons

We next determined whether the odor-evoked activity of the MBON dendrites in the β′ lobe was modified by training. Flies were trained using either an appetitive or an aversive conditioning protocol and were subsequently captured and prepared for live-imaging of odor-evoked activity within a window of 1–2 hr after training. Importantly, blocking M4β′/MBON-β′2mp and M6/MBON- γ5β′2a neurons impaired both appetitive and aversive memory 2 hr after training (Figures 3E, 3F, and S5 for permissive temperature controls). We monitored the calcium responses evoked in MBON dendrites in the β′ lobe (example traces shown in Figures 3G and 3H) by exposing the flies to either the odor that had been previously paired with sugar reward or electric-shock punishment (the CS+), or to the non-reinforced odor (the CS−). We also compared the odor-evoked responses in flies that were mock-trained—subjected to the full conditioning regimen of odor presentation but without reinforcement delivery. In addition, we performed both the aversive and appetitive protocols using either MCH as the CS+ and OCT as the CS−, or OCT as the CS+ and MCH as the CS−. Strikingly, in both appetitive conditioning experiments the response to the CS+ relative to the CS− was decreased when compared to the responses in mock trained flies (Figures 3I and 3K). Moreover, this relationship was reversed in each experiment following aversive training, with the relative CS+ to CS− evoked response being increased when compared to the responses in mock trained flies (Figures 3J and 3L). These data suggest that the relative odor drive to the MBON dendrites in the β′ lobe is bidirectionally tuned by olfactory conditioning, and they are consistent with the relative conditioned odor drive being depressed by appetitive learning and potentiated by aversive learning.

Direct Manipulation of M4/6 Neurons Can Mimic Learning in Naive Flies

We reasoned that if a reduced conditioned-odor drive to MBON dendrites in the β′ lobe was an important element of appetitive learning, we might be able to mimic conditioned approach by directly inhibiting the M4/6 neurons. In general, naive flies are repelled by high concentrations of odor when presented in a choice with a clean air stream (Tully and Quinn, 1985; Heimbeck et al., 2001). We therefore used UAS-*shi*^{ts1} to test whether M4/6 neuron block altered naive odor avoidance behavior. Control flies that were either heterozygous for the R21D02-GAL4, VT1211-GAL4, or the UAS-*shi*^{ts1} effector transgene showed robust avoidance of MCH when presented at 100-, 1,000-, and 4,000-fold dilutions. Strikingly, at the two lower concentrations, blocking M4/6 neurons converted naive odor avoidance behavior into significant odor approach (Figure 4A). A similar abolishment and reversal of avoidance was also observed with OCT, although the effective concentration range appears to be different (Figure 4B). Interestingly, blocking only the M6/MBON-γ5β′2a neurons blunted the aversion but did not induce behavioral reversal (Figures 4C and 4D), indicating that the M4β′/MBON-β′2mp neurons play a particularly prominent role. Furthermore, no significant effects on odor avoidance were observed when the same flies were tested at the permissive temperature (Figure S8) or when blocking the previously described MB-V2α/MBON-α2sc and MB-V2α′/MBON-α′3 (Séjourné et al., 2011; Aso et al., 2014) or MB-V3/MBON-α3 (Pai et al., 2013; Plaçais et al., 2013; Aso et al., 2014) output neurons that are dendritic to the vertical lobes of the mushroom body (Figures 4C and 4D). We speculate that the loss of the phenotype at high MCH concentration reflects either a ceiling effect or a significant role for the LH. Nevertheless, these data indicate that the observed behavioral reversals are specific to blocking M4β′/MBON-β′2mp and M6/MBON- γ5β′2a neurons and that inhibiting these output pathways can convert odor avoidance into odor attraction in a manner that reflects appetitive conditioning.

Our imaging data also indicate that the relative CS+ to CS− odor drive to MBON dendrites in the β′ lobe is increased after aversive conditioning. We therefore tested whether activation of M4/6 neurons promoted avoidance behavior. We expressed a UAS-*ReaChR* red-light-activated channelrhodopsin transgene (Inagaki et al., 2014) in M4/6 neurons using R21D02-GAL4 and allowed flies to choose between an unlit arm and a red-light-illuminated arm in a T-maze. Whereas all control flies distributed evenly between the tubes, a significant fraction of R21D02;*ReaChR* flies avoided the illuminated arm (Figure 4E), consistent with M4/6 neuron activity driving avoidance behavior. Therefore, both the imaging of odor-evoked responses after training and the behavioral experiments reveal bidirectional phenotypes that are consistent with the KC-M4/6 junction being a key site that provides direction to odor-driven behavior after aversive and appetitive training.

DISCUSSION

Many prior studies, including our own, have concluded that mushroom body neurons are dispensable for naive odor-driven behavior and subsets are either required or are dispensable for particular memory functions (Heimbeck et al., 2001; Heisenberg et al., 1985; Dubnau et al., 2001; McGuire et al., 2001; Schwaerzel et al., 2002; Krashes et al., 2007; Cervantes-Sandoval et al., 2013; Isabel et al., 2004; Huang et al., 2012; Perisse et al., 2013; Xie et al., 2013). However, these experiments simultaneously blocked all the outputs from a given population of KCs using cell-wide expression of *shi*^{ts1}. Our results here suggest that these models should be reconsidered. Blocking the specific M4β/MBON-β2β′2a, M4β′/MBON-β′2mp, and M6/MBON-γ5β′2a output from the mushroom body, as opposed to blocking all outputs, has a radical effect on naive odor-driven behavior. We propose that ordinarily, in naive flies, the multiple mushroom body output channels are ultimately pooled and contribute a net zero to odor-driven behavior. Therefore, if one uses a mushroom body neuron-driven UAS-*shi*^{ts1} that simultaneously blocks all outputs, there is no apparent effect on naive behavior. If, however, one blocks only one channel, or alters its efficacy by learning, the odor-driven behavior can be changed. A similar

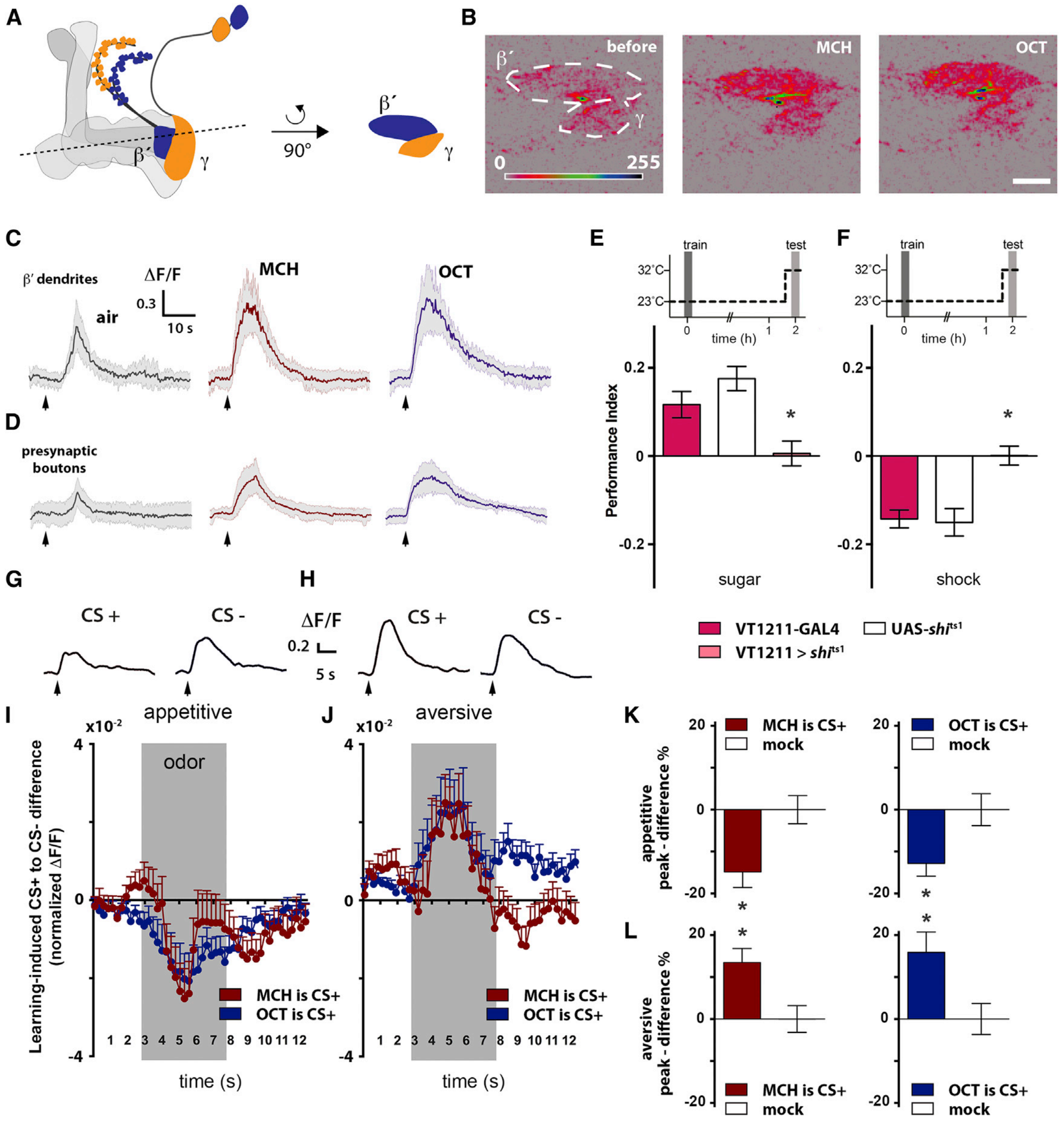

Figure 3. Odor-Evoked Responses in MBON Dendrites in the β′ Lobe Are Bidirectionally Altered by Conditioning

(A) Schematic of the imaging plane and area of interest of the M4β′/MBON-β′2mp neuron.

(B) Example pseudocolored traces of calcium transients measured in the MBON dendrites in the β′ lobe in a naive fly exposed to MCH or OCT, the odors used in conditioning. Scale bar is 10 μm.

(C and D) Time courses of odor-evoked GCaMP responses (ΔF/F) collected at the level of the M4β′ neuron dendrites (C) (n = 18, nine animals) or presynaptic boutons (D) (n = 9, nine animals). Traces represent mean odor responses (solid line) and standard deviation (gray shading). Arrows indicate onset of odor presentation.

(E and F) Blocking M4β′/MBON-β′2mp and M6/MBON-γ5β′2a neurons significantly impairs 2 hr appetitive (E) ($n \geq 16$, $p < 0.05$) and aversive memory retrieval (F) ($n \geq 19$, $p < 0.05$). Statistics are one-way ANOVA followed by a Tukey's HSD post-hoc test. Data shown are the mean ± SEM.

(G and H) Single example traces of calcium transients evoked by trained odors recorded from MBON dendrites in the β′ lobe 1–2 hr after (G) appetitive and (H) aversive conditioning. In these examples CS+ is the MCH responses and CS− is the OCT response. Arrows indicate onset of odor presentation.

(I) Difference of responses evoked by the CS+ (MCH in red, OCT in blue) and CS− following appetitive training relative to the mean transients of mock trained flies (also see Figures S6 and S7).

(J) Difference of responses evoked by the CS+ (MCH in red, OCT in blue) and CS− following aversive training. Shock training shifts the curve toward a relative increase of the CS+ response, while sugar training shifts the curve in the opposite direction. Data shown are the mean ± SEM. Light gray boxes indicate the time of the odor exposure.

(legend continued on next page)

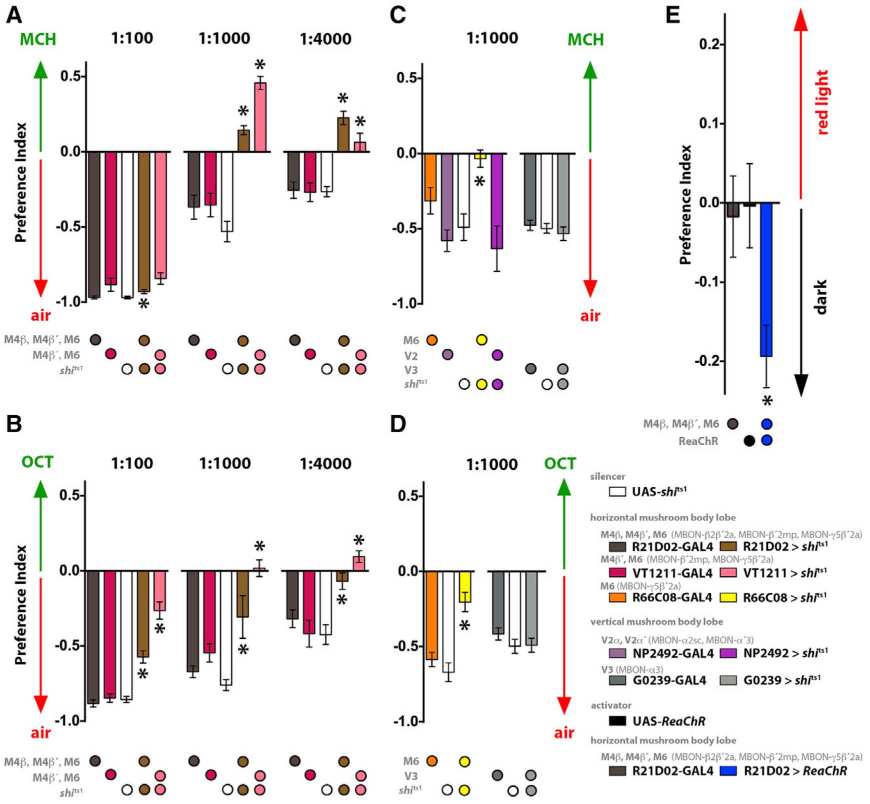

Figure 4. Blocking M4/6 Neurons Mimics Appetitive Conditioning by Converting Naive Odor Avoidance into Attraction

(A) Blocking M4/6 neurons in naive flies with either R21D02 (M4β/MBON-β2β′2a, M4β′/MBON-β′2mp, and M6/MBON-γ5β′2a) or VT1211-driven (M4β′/MBON-β′2mp and M6/MBON-γ5β′2a) UAS-*shi*^{ts1} reverses the behavioral response to 1:1,000 and 1:4,000 MCH. Robust avoidance behavior is converted into approach behavior (1:1,000: $n \geq 8$, $p < 0.05$; 1:4,000: $n \geq 14$, $p < 0.05$). R21D02; UAS-*shi*^{ts1} flies showed a significant decrease in avoidance of 1:100 MCH ($n = 8$, $p < 0.05$), but VT1211; UAS-*shi*^{ts1} flies were not significantly different from VT1211 alone ($n = 8$, $p > 0.05$).

(B) Blocking M4/6 neurons in naive flies also impairs or reverses OCT avoidance (1:100: $n \geq 8$, $p < 0.05$; 1:1,000: $n \geq 11$, $p < 0.05$; 1:4,000: $n = 8$, $p < 0.05$ for VT1211;*shi*^{ts1} and $p > 0.05$ for R21DO2;*shi*^{ts1}).

(C and D) Blocking M6/MBON-γ5β′2a neurons in naive flies with R66C08-driven UAS-*shi*^{ts1} reduces avoidance to MCH (C) (1:1,000 dilution; $n \geq 10$, $p < 0.05$) and OCT (D) (1:1,000 dilution; $n = 6$, $p < 0.05$). Blocking the vertical α-lobe output MB-V3/MBON-α3 ($n \geq 5$, $p < 0.05$) using G0239-GAL4 does not impair naive MCH or OCT avoidance. Blocking the MB-V2α/MBON-α2sc and MB-V2α′/MBON-α′3 vertical α- and α′-lobe outputs ($n \geq 6$, $p < 0.05$) using NP2492-GAL4 does not impair MCH avoidance.

(E) Flies avoid optogenetic activation of M4β/MBON-β2β′2a, M4β′/MBON-β′2mp, and M6/MBON-γ5β′2a neurons ($n \geq 13$, $p < 0.05$). Data are the mean ± SEM. Asterisks denote $p < 0.05$; all statistics are one-way ANOVA followed by a Tukey's HSD post-hoc test.

logic could also account for why we observe clear memory retrieval defects when blocking M4β′/MBON-β′2mp and M6/MBON-γ5β′2a neurons that presumably pool outputs from the tip of the γ and β′ lobe, yet blocking all α′β′ neuron outputs did not demonstrably disrupt later memory retrieval (Krashes et al., 2007; Krashes and Waddell, 2008). Others have shown a role for α′β′ neuron output to retrieve earlier forms of memory (Wang et al., 2008; Cervantes-Sandoval et al., 2013).

Both our physiological and behavioral results are consistent with a depression of the M4β′/MBON-β′2mp and M6/MBON-γ5β′2a output being sufficient to code learned approach. Learning-related plasticity has been reported at the β-lobe outputs in both bees (Okada et al., 2007) and locusts (Cassenaer and Laurent, 2012), although the importance of these synaptic connections in the behavior of these insects is not known. At this stage we cannot be sure that our observed decrease in the relative odor drive reflects plasticity of the synapses between odor-specific KCs and the M4/6 neurons. However, it seems plausible, because this synaptic junction is addressed by the relevant rewarding dopaminergic neurons (Burke et al., 2012). Given that blocking M4β′/MBON-β′2mp and M6/MBON-γ5β′2a neurons converts avoidance to approach, other mushroom body output channels, perhaps some of which lie on the vertical α-lobe projection (Séjourné et al., 2011; Plaçais et al., 2013), must drive the approach behavior. It is therefore conceivable that a similar plasticity of odor drive to these putative approach outputs could be critical for aversive conditioning. Such an idea is consistent with several prior reports of aversive memory traces that are specific to the vertical α-branch of the mushroom body (Yu et al., 2005, 2006; Cervantes-Sandoval and Davis, 2012). In addition, aversive learning has been reported to depress odor drive in the vertical lobe of downstream MB-V2α/MBON-α2sc and MB-V2α′/MBON-α′3 neurons (Séjourné et al., 2011; Aso et al., 2014) and to potentiate odor drive of MB-V3/MBON-α3 output neurons (Pai et al., 2013; although Plaçais et al. [2013] reported potentiation after appetitive learning). However, it is notable that blocking either the MB-V2α/MBON-α2sc and MB-V2α′/MBON-α′3 neurons or MB-V3/MBON-α3 neurons

(K and L) Bar graphs illustrate peak ± 0.5 s values of the odor response difference curves for trained and mock trained animals expressed as a percentage difference to the mean of the mock (see Experimental Procedures, Equation 2), for (K) appetitive or (L) aversive paradigms. Data are mean ± SEM; for MCH as CS+: n (appetitively trained) = 22, 11 animals, n (mock) = 19, 11 animals, $p < 0.05$; n (aversively trained) = 24, 13 animals, n (mock) = 19, 11 animals, $p < 0.05$; for OCT as CS+: n (appetitively trained) = 59, 32 animals, n (mock) = 58, 31 animals, $p < 0.05$; n (aversively trained) = 37, 20 animals, n (mock) = 29, 16 animals, $p < 0.05$; statistics are Mann-Whitney U-test.

did not affect naive odor avoidance behavior in our experiments or those of others (Séjourné et al., 2011; Pai et al., 2013; Plaçais et al., 2013). Therefore, although MB-V2α/MBON-α2sc, MB-V2α′/MBON-α′3, and MB-V3/MBON-α3 neurons are required for memory expression, it is not currently known which reinforcing neurons address MB-V2α/MBON-α2sc, MB-V2α′/MBON-α′3, and MB-V3/MBON-α3 connections and how these outputs specifically contribute to odor-guided behavior.

Our physiological analyses suggest bidirectional plasticity of odor-evoked responses, with aversive learning increasing the relative conditioned odor drive to the M4β′/MBON-β′2mp neurons. This could account for why output from M4/6 neurons is also required for expression of aversive memory. Moreover, whereas blocking the M4β/MBON-β2β′2a, M4β′/MBON-β′2mp, and M6/MBON-γ5β′2a neurons converts odor avoidance into approach, activation of M4β/MBON-β2β′2a, M4β′/MBON-β′2mp, and M6/MBON-γ5β′2a neurons drives avoidance. It therefore seems likely that plasticity of the relative odor drive to M4β′/MBON-β′2mp neurons is also part of the aversive memory engram. Again, we do not know that the increased odor drive after training reflects synaptic potentiation between odor-specific KCs and the M4β′/MBON-β′2mp neurons. Increased odor drive to M4β′/MBON-β′2mp neurons could, for example, also result from plasticity elsewhere in the KCs that enhances signal propagation along the horizontal KC arbor. Nevertheless, the MB-M3 dopaminergic neurons that are required to reinforce aversive memory also innervate the tips of the β and β′ lobe (Aso et al., 2012). In addition, a recent study reported that aversive learning specifically decreased unconditioned odor-evoked neurotransmission from the γ neurons (Zhang and Roman, 2013), a result that presumably would mirror a relative increase in the response to the reinforced odor. Lastly, aversive conditioning using relative shock intensity utilizes the rewarding dopaminergic neurons (Perisse et al., 2013) that occupy the same zones on the mushroom body as the M4β′/MBON-β′2mp and M6/MBON-γ5β′2a neuron dendrites. With the caveat that GRASP is only an indicator of proximity, our anatomical studies suggest that dendrites of rewarding dopaminergic neurons may connect to the M4β′/MBON-β′2mp and M6/MBON-γ5β′2a neuron presynaptic terminals, forming a potential feedback or forward loop that could serve such a relative-judgment function.

It is perhaps noteworthy that KC outputs in the vertical lobe are onto excitatory cholinergic MB-V2α/MBON-α2sc and MB-V2α′/MBON-α′3 (Séjourné et al., 2011) and MB-V3/MBON-α3 (Pai et al., 2013; Plaçais et al., 2013) neurons, whereas the horizontal outputs are onto glutamatergic, potentially inhibitory (Liu and Wilson, 2013), M4β/MBON-β2β′2a, M4β′/MBON-β′2mp, and M6/MBON-γ5β′2a neurons. This suggests that distinct signaling modes may be driven from the bifurcated collaterals of KCs. It will be crucial to understand how these outputs from the different branches, and those from discrete lobes, are ultimately pooled to guide appropriate behavior.

EXPERIMENTAL PROCEDURES

Fly Strains

All flies were reared on standard cornmeal-agar food at either 25°C or 18°C. The driver lines used were R21D02-GAL4, R66D08-GAL4, R48B04-LexA (Jenett et al., 2012; Lin et al., 2014), R58E02-LexA (Liu et al., 2012), G0239-GAL4 (Pai et al., 2013), NP2492-GAL4 (Séjourné et al., 2011), and VT1211-GAL4 (Bidaye et al., 2014). GAL4 driver lines were crossed to UAS-*shi*^{ts1} (Kitamoto, 2001) or UAS-*ReaChR* (Inagaki et al., 2014). GAL4 driver lines, UAS-*shi*^{ts1}, or UAS-*ReaChR* were crossed to Canton-S flies as controls. For anatomy, driver lines were combined with UAS-mCD8-GFP (Lee and Luo, 1999), 247-LexA (Pitman et al., 2011), LexAop-mCD2-mRFP (Lai and Lee, 2006), UAS-DenMark-mRFP (Nicolaï et al., 2010), or UAS-GFP-Syd-1 (Owald et al., 2010). GRASP experiments were performed as described (Gordon and Scott, 2009; Pitman et al., 2011).

Confocal Imaging and Immunostaining

All confocal images were acquired on a Leica SP5 at manually adjusted laser intensity and gain. Brains were dissected on ice and fixed in 4% paraformaldehyde. For native fluorophore imaging, samples were incubated and washed in PBT (0.1% Triton) and PBS before mounting. For immunostainings, brains were incubated in PBT (0.1% Triton) supplemented with a rabbit anti-DVGlut primary antibody (Mahr and Aberle, 2006) (1:500 dilution), followed by incubation with secondary antibodies (Alexa 647, Sigma).

Two-Photon Calcium Imaging

We imaged 3- to 8-day-old UAS-GCaMP6m; VT1211-GAL4 female flies 1–2 hr after training. Flies were trained using either 4-MCH or 3-OCT as the CS+ and the reciprocal odor as the CS− in a T-maze (see below). Mock trained flies were exposed to MCH and OCT with no sugar or shock reinforcement. For imaging, flies were briefly anesthetized < 10 s on ice and mounted in a custom-made chamber. The head capsule was opened under room temperature sugar-free HL3-like saline (Yoshihara, 2012). The legs and proboscis were immobilized with wax. Fluorescence was excited using 70 fs pulses, 80 MHz repetition rate, centered on 910 nm generated by a Ti-Sapphire laser (Chameleon Ultra II, Coherent). Images of 256 × 128 pixels were acquired at 11.5 Hz using two-photon microscopy (Scientifica) with a 40X, 0.8 NA water-immersion objective, controlled by ScanImage 3.8 software (Pologruto et al., 2003). Odors were delivered on a clean air carrier stream using a custom-designed system (Shang et al., 2007), which also synchronizes the timing of odor delivery and the two-photon image acquisition. Two-photon fluorescence images were manually segmented using ImageJ. Movement of the animal was small enough such that images did not require registration. The fluorescence over the defined region of interest was summed at each frame to yield one fluorescence trace, F(*t*). Where possible, each hemisphere was separately evaluated and treated as an independent "n." All subsequent analyses utilized custom-written Matlab routines. Flies were exposed to two consecutive 5 s clean air puffs with 30 s intervals. First responses were discarded and second responses were defined as the "no odor response." After brief rest, flies were exposed to 5 s MCH (air stream passing over 10^{-2} odor dilution in mineral oil, and then further blended 1:9 with a clean air stream), then 30 s clean air, followed by 5 s OCT pulse. This odor stimulation protocol was delivered twice. Baseline fluorescence (F) corresponds to the average fluorescence signal across an 8 s window starting 9 s after scan onset and terminating 3 s before the first air or odor exposure. The baseline was then used to compute the relative change in fluorescence (ΔF(*t*)/F = (F(*t*) − F)/F). Responses were determined to start 2.5 s after the instrumentation odor delivery command and to end within 12.5 s. This delayed onset accounts for the computational, electronic, mechanical, and fluid flow lag. The response curves were normalized and averaged over the two paired odor presentations:

$$CS_n^{+/-}(t) = \frac{1}{2}\sum_{i=1}^{2} \frac{CS_{n,i}^{+/-}(t)}{\int_0^{12.5}\left(\frac{CS_{n,i}^{+}(t)+CS_{n,i}^{-}(t)}{2}\right)dt} \quad \text{(Equation 1)}$$

$CS_{n,i}^{+/-}(t)$ are the ΔF(*t*)/F response curves of the "n"th experiment to the "i"th odor stimulation protocol. The normalization factor was chosen to be the average of the total CS+ and CS− response to avoid bias toward one or the other and was calculated as the sum over the acquisition time points of the ΔF(*t*)/F curves multiplied by the sampling interval. We then computed the odor response difference for each n, $D_n(t) = CS_n^+ - CS_n^-$. To quantify

the difference between the trained and mock groups, the area under the peak of each curve (defined as 5 ± 0.5 s after odor delivery) was computed and expressed as a percentage difference to the mean of the mock response curves:

$$Peak_n = 100 \times \frac{\int_{4.5}^{5.5} D_n(t)dt - \langle D \rangle_{mock}}{\langle D \rangle_{mock}} \quad \text{(Equation 2)}$$

$< D >_{mock}$ is the mean of the odor response difference curves in the corresponding mock group:

$$\langle D \rangle_{mock} = \frac{1}{N_{mock}} \sum_{j=1}^{N_{mock}} \int_{4.5}^{5.5} D_j(t)dt \quad \text{(Equation 3)}$$

where the summation is over the experiments in the relevant mock group and N_{mock} is the number of experiments in that group. We note that by inspection of Equation 2, the average of the $Peak_n$ values for each mock group will be zero. The *Peak* values obtained from each trained group were compared with those of the corresponding mock group using the Mann-Whitney U-test (see Figure S6 for normalized odor response traces and Figure S7 for analysis overview). The learning-induced difference curve, L(t), is the difference between the mean ± SEM of the $D_n(t)$ curves of the trained and corresponding mock groups. The errors were combined in the usual way, i.e., error in $L(t) = \sqrt{SEM(t)^2_{trained} + SEM(t)^2_{mock}}$. Graphs were created in Prism 6 (GraphPad Software).

Behavior

For appetitive and aversive memory testing, flies were reared at 25°C and 4- to 9-day-old mixed-sex populations were tested together in all experiments. Flies were starved for 21–24 h prior to appetitive training (Krashes and Waddell, 2008). Flies were also starved after training for 2 hr and 24 hr memory testing. Aversive and appetitive training was performed as described (Perisse et al., 2013). Briefly, for appetitive conditioning flies were exposed to the CS− for 2 min followed by 30 s of air and then to the CS+ in the presence of dry sucrose for 2 min. For aversive conditioning flies were exposed to the CS+ for 1 min with twelve 90 V electric shocks at 5 s intervals followed by 45 s of air and the CS− for 1 min. For testing flies were given 2 min to choose between the CS+ and CS− in a T-maze. Performance index (PI) was calculated as the number of flies approaching (appetitive memory) or avoiding (aversive memory) the conditioned odor, minus the number of flies going the other direction, divided by the total number of flies in the experiment. A single PI value is the average score from flies of the identical genotype tested with the reciprocal reinforced/non-reinforced odor combination (Tully and Quinn, 1985; Perisse et al., 2013). Permissive temperature was 23°C and restrictive 32°C. Odor dilutions were adjusted between experiments and odor batches to minimize bias (MCH 5–8 μl in 8 ml mineral oil and OCT 7–8 μl in 8 ml mineral oil). All memory experiments utilized a transgenic line with UAS-*shibire*^{ts1} on the X and III chromosome.

To assay naive odor choice, 5-day-old flies were starved for 21–24 hr prior to testing. Flies were allowed to choose between MCH or OCT (1:100, 1:1,000, or 1:4,000 dilution in mineral oil) and mineral oil-suffused air streams for 2 min. Preference index was calculated as the number of flies approaching the odor minus the number approaching mineral oil, divided by the total number of flies in the experiment. All naive odor choice experiments utilized a transgenic line with UAS-*shi*^{ts1} on the III chromosome, and crosses were reared at 18°C. One "n" corresponds to a single test trial.

For optogenetic experiments flies were kept on food supplemented with 1 mM retinal for 2 days prior to testing. Three high-power LEDs (700 mA, centered at 630 nm) were mounted on one arm of the T-maze and triggered for 100 ms at 5 Hz. Flies were given 1 min to choose between the illuminated or non-illuminated arm.

Statistical Analysis

Data were analyzed using Matlab and Prism 6. All behavioral data were analyzed with a one-way ANOVA followed by a Tukey's honestly significant difference (HSD) post-hoc test. Imaging data were analyzed using a Mann-Whitney U-test. Definition of statistical significance is set at $p < 0.05$.

SUPPLEMENTAL INFORMATION

Supplemental Information includes eight figures and three movies and can be found with this article online at http://dx.doi.org/10.1016/j.neuron.2015.03.025.

AUTHOR CONTRIBUTIONS

D.O. and S.W. conceived this project and designed all experiments. J.F., G.D., E.P., and D.O. performed all behavioral experiments. Live-imaging was performed by D.O. using custom apparatus and software designed by D.O. and C.B.T. and constructed and programmed by C.B.T. Imaging data were analyzed by D.O and C.B.T. using software programmed by C.B.T. and designed by D.O. and C.B.T. GAL4 lines were visually screened and selected by W.H. and D.O. Anatomical data were produced by W.H. and D.O. The manuscript was written by S.W. and D.O.

ACKNOWLEDGMENTS

We thank Gero Miesenböck and members of the Waddell and Miesenböck labs for support and discussion and Paola Cognigni for help with the ReaChR experiments. We thank H. Aberle for the DVGlut antibody, G. Rubin and the Janelia Farm Project, B. Dickson and the VDRC, and the Bloomington stock center for fly lines. D.O. was supported by an EMBO Long-Term Fellowship and a Sir Henry Wellcome Postdoctoral Fellowship. E.P. thanks the Philippe and Bettencourt-Schueller Foundations. S.W. is funded by a Wellcome Trust Senior Research Fellowship in the Basic Biomedical Sciences and by funds from the Gatsby Charitable Foundation and Oxford Martin School.

Received: October 28, 2014
Revised: December 16, 2014
Accepted: February 20, 2015
Published: April 9, 2015

REFERENCES

Aso, Y., Herb, A., Ogueta, M., Siwanowicz, I., Templier, T., Friedrich, A.B., Ito, K., Scholz, H., and Tanimoto, H. (2012). Three dopamine pathways induce aversive odor memories with different stability. PLoS Genet. *8*, e1002768.

Aso, Y., Hattori, D., Yu, Y., Johnston, R.M., Iyer, N.A., Ngo, T.T., Dionne, H., Abbott, L.F., Axel, R., Tanimoto, H., and Rubin, G.M. (2014). The neuronal architecture of the mushroom body provides a logic for associative learning. eLife *3*, e04577.

Bidaye, S.S., Machacek, C., Wu, Y., and Dickson, B.J. (2014). Neuronal control of Drosophila walking direction. Science *344*, 97–101.

Burke, C.J., Huetteroth, W., Owald, D., Perisse, E., Krashes, M.J., Das, G., Gohl, D., Silies, M., Certel, S., and Waddell, S. (2012). Layered reward signalling through octopamine and dopamine in Drosophila. Nature *492*, 433–437.

Busto, G.U., Cervantes-Sandoval, I., and Davis, R.L. (2010). Olfactory learning in Drosophila. Physiology (Bethesda) *25*, 338–346.

Cassenaer, S., and Laurent, G. (2012). Conditional modulation of spike-timing-dependent plasticity for olfactory learning. Nature *482*, 47–52.

Cervantes-Sandoval, I., and Davis, R.L. (2012). Distinct traces for appetitive versus aversive olfactory memories in DPM neurons of Drosophila. Curr. Biol. *22*, 1247–1252.

Cervantes-Sandoval, I., Martin-Peña, A., Berry, J.A., and Davis, R.L. (2013). System-like consolidation of olfactory memories in Drosophila. J. Neurosci. *33*, 9846–9854.

Chen, T.W., Wardill, T.J., Sun, Y., Pulver, S.R., Renninger, S.L., Baohan, A., Schreiter, E.R., Kerr, R.A., Orger, M.B., Jayaraman, V., et al. (2013). Ultrasensitive fluorescent proteins for imaging neuronal activity. Nature *499*, 295–300.

Claridge-Chang, A., Roorda, R.D., Vrontou, E., Sjulson, L., Li, H., Hirsh, J., and Miesenböck, G. (2009). Writing memories with light-addressable reinforcement circuitry. Cell *139*, 405–415.

CellPress

Couto, A., Alenius, M., and Dickson, B.J. (2005). Molecular, anatomical, and functional organization of the Drosophila olfactory system. Curr. Biol. *15*, 1535–1547.

Daniels, R.W., Gelfand, M.V., Collins, C.A., and DiAntonio, A. (2008). Visualizing glutamatergic cell bodies and synapses in Drosophila larval and adult CNS. J. Comp. Neurol. *508*, 131–152.

de Bruyne, M., Clyne, P.J., and Carlson, J.R. (1999). Odor coding in a model olfactory organ: the Drosophila maxillary palp. J. Neurosci. *19*, 4520–4532.

de Bruyne, M., Foster, K., and Carlson, J.R. (2001). Odor coding in the Drosophila antenna. Neuron *30*, 537–552.

Dobritsa, A.A., van der Goes van Naters, W., Warr, C.G., Steinbrecht, R.A., and Carlson, J.R. (2003). Integrating the molecular and cellular basis of odor coding in the Drosophila antenna. Neuron *37*, 827–841.

Dubnau, J., and Chiang, A.S. (2013). Systems memory consolidation in Drosophila. Curr. Opin. Neurobiol. *23*, 84–91.

Dubnau, J., Grady, L., Kitamoto, T., and Tully, T. (2001). Disruption of neurotransmission in Drosophila mushroom body blocks retrieval but not acquisition of memory. Nature *411*, 476–480.

Feinberg, E.H., Vanhoven, M.K., Bendesky, A., Wang, G., Fetter, R.D., Shen, K., and Bargmann, C.I. (2008). GFP Reconstitution Across Synaptic Partners (GRASP) defines cell contacts and synapses in living nervous systems. Neuron *57*, 353–363.

Fişek, M., and Wilson, R.I. (2014). Stereotyped connectivity and computations in higher-order olfactory neurons. Nat. Neurosci. *17*, 280–288.

Fishilevich, E., and Vosshall, L.B. (2005). Genetic and functional subdivision of the Drosophila antennal lobe. Curr. Biol. *15*, 1548–1553.

Gao, Q., Yuan, B., and Chess, A. (2000). Convergent projections of Drosophila olfactory neurons to specific glomeruli in the antennal lobe. Nat. Neurosci. *3*, 780–785.

Gordon, M.D., and Scott, K. (2009). Motor control in a Drosophila taste circuit. Neuron *61*, 373–384.

Hallem, E.A., and Carlson, J.R. (2004). The odor coding system of Drosophila. Trends Genet. *20*, 453–459.

Hallem, E.A., and Carlson, J.R. (2006). Coding of odors by a receptor repertoire. Cell *125*, 143–160.

Heimbeck, G., Bugnon, V., Gendre, N., Keller, A., and Stocker, R.F. (2001). A central neural circuit for experience-independent olfactory and courtship behavior in Drosophila melanogaster. Proc. Natl. Acad. Sci. USA *98*, 15336–15341.

Heisenberg, M. (2003). Mushroom body memoir: from maps to models. Nat. Rev. Neurosci. *4*, 266–275.

Heisenberg, M., Borst, A., Wagner, S., and Byers, D. (1985). Drosophila mushroom body mutants are deficient in olfactory learning. J. Neurogenet. *2*, 1–30.

Honegger, K.S., Campbell, R.A., and Turner, G.C. (2011). Cellular-resolution population imaging reveals robust sparse coding in the Drosophila mushroom body. J. Neurosci. *31*, 11772–11785.

Huang, C., Zheng, X., Zhao, H., Li, M., Wang, P., Xie, Z., Wang, L., and Zhong, Y. (2012). A permissive role of mushroom body α/β core neurons in long-term memory consolidation in Drosophila. Curr. Biol. *22*, 1981–1989.

Huber, F. (1967). Central control of movements and behavior of invertebrates. In Invertebrate Nervous Systems, C.A.G. Wiersma, ed. (Chicago: University of Chicago Press), pp. 333–351.

Inagaki, H.K., Jung, Y., Hoopfer, E.D., Wong, A.M., Mishra, N., Lin, J.Y., Tsien, R.Y., and Anderson, D.J. (2014). Optogenetic control of Drosophila using a red-shifted channelrhodopsin reveals experience-dependent influences on courtship. Nat. Methods *11*, 325–332.

Isabel, G., Pascual, A., and Preat, T. (2004). Exclusive consolidated memory phases in Drosophila. Science *304*, 1024–1027.

Ito, K., Shinomiya, K., Ito, M., Armstrong, J.D., Boyan, G., Hartenstein, V., Harzsch, S., Heisenberg, M., Homberg, U., Jenett, A., et al.; Insect Brain Name Working Group (2014). A systematic nomenclature for the insect brain. Neuron *81*, 755–765.

Jefferis, G.S., Marin, E.C., Stocker, R.F., and Luo, L. (2001). Target neuron prespecification in the olfactory map of Drosophila. Nature *414*, 204–208.

Jenett, A., Rubin, G.M., Ngo, T.T., Shepherd, D., Murphy, C., Dionne, H., Pfeiffer, B.D., Cavallaro, A., Hall, D., Jeter, J., et al. (2012). A GAL4-driver line resource for Drosophila neurobiology. Cell Rep. *2*, 991–1001.

Kazama, H., and Wilson, R.I. (2008). Homeostatic matching and nonlinear amplification at identified central synapses. Neuron *58*, 401–413.

Kim, Y.C., Lee, H.G., and Han, K.A. (2007). D1 dopamine receptor dDA1 is required in the mushroom body neurons for aversive and appetitive learning in Drosophila. J. Neurosci. *27*, 7640–7647.

Kitamoto, T. (2001). Conditional modification of behavior in Drosophila by targeted expression of a temperature-sensitive shibire allele in defined neurons. J. Neurobiol. *47*, 81–92.

Krashes, M.J., and Waddell, S. (2008). Rapid consolidation to a radish and protein synthesis-dependent long-term memory after single-session appetitive olfactory conditioning in Drosophila. J. Neurosci. *28*, 3103–3113.

Krashes, M.J., Keene, A.C., Leung, B., Armstrong, J.D., and Waddell, S. (2007). Sequential use of mushroom body neuron subsets during drosophila odor memory processing. Neuron *53*, 103–115.

Lai, S.L., and Lee, T. (2006). Genetic mosaic with dual binary transcriptional systems in Drosophila. Nat. Neurosci. *9*, 703–709.

Lee, T., and Luo, L. (1999). Mosaic analysis with a repressible cell marker for studies of gene function in neuronal morphogenesis. Neuron *22*, 451–461.

Liang, L., Li, Y., Potter, C.J., Yizhar, O., Deisseroth, K., Tsien, R.W., and Luo, L. (2013). GABAergic projection neurons route selective olfactory inputs to specific higher-order neurons. Neuron *79*, 917–931.

Lin, S., Owald, D., Chandra, V., Talbot, C., Huetteroth, W., and Waddell, S. (2014). Neural correlates of water reward in thirsty *Drosophila*. Nat. Neurosci. *17*, 1536–1542.

Liu, W.W., and Wilson, R.I. (2013). Glutamate is an inhibitory neurotransmitter in the Drosophila olfactory system. Proc. Natl. Acad. Sci. USA *110*, 10294–10299.

Liu, C., Plaçais, P.Y., Yamagata, N., Pfeiffer, B.D., Aso, Y., Friedrich, A.B., Siwanowicz, I., Rubin, G.M., Preat, T., and Tanimoto, H. (2012). A subset of dopamine neurons signals reward for odour memory in Drosophila. Nature *488*, 512–516.

Mahr, A., and Aberle, H. (2006). The expression pattern of the Drosophila vesicular glutamate transporter: a marker protein for motoneurons and glutamatergic centers in the brain. Gene Expr. Patterns *6*, 299–309.

Mao, Z., and Davis, R.L. (2009). Eight different types of dopaminergic neurons innervate the Drosophila mushroom body neuropil: anatomical and physiological heterogeneity. Front Neural Circuits *3*, 5.

Martin, J.R., Ernst, R., and Heisenberg, M. (1998). Mushroom bodies suppress locomotor activity in Drosophila melanogaster. Learn. Mem. *5*, 179–191.

Masse, N.Y., Turner, G.C., and Jefferis, G.S. (2009). Olfactory information processing in Drosophila. Curr. Biol. *19*, R700–R713.

McGuire, S.E., Le, P.T., and Davis, R.L. (2001). The role of Drosophila mushroom body signaling in olfactory memory. Science *293*, 1330–1333.

Nicolaï, L.J., Ramaekers, A., Raemaekers, T., Drozdzecki, A., Mauss, A.S., Yan, J., Landgraf, M., Annaert, W., and Hassan, B.A. (2010). Genetically encoded dendritic marker sheds light on neuronal connectivity in Drosophila. Proc. Natl. Acad. Sci. USA *107*, 20553–20558.

Okada, R., Rybak, J., Manz, G., and Menzel, R. (2007). Learning-related plasticity in PE1 and other mushroom body-extrinsic neurons in the honeybee brain. J. Neurosci. *27*, 11736–11747.

Olsen, S.R., Bhandawat, V., and Wilson, R.I. (2010). Divisive normalization in olfactory population codes. Neuron *66*, 287–299.

Owald, D., Fouquet, W., Schmidt, M., Wichmann, C., Mertel, S., Depner, H., Christiansen, F., Zube, C., Quentin, C., Körner, J., et al. (2010). A Syd-1 homologue regulates pre- and postsynaptic maturation in Drosophila. J. Cell Biol. *188*, 565–579.

Pai, T.P., Chen, C.C., Lin, H.H., Chin, A.L., Lai, J.S., Lee, P.T., Tully, T., and Chiang, A.S. (2013). Drosophila ORB protein in two mushroom body output neurons is necessary for long-term memory formation. Proc. Natl. Acad. Sci. USA *110*, 7898–7903.

Parnas, M., Lin, A.C., Huetteroth, W., and Miesenböck, G. (2013). Odor discrimination in Drosophila: from neural population codes to behavior. Neuron *79*, 932–944.

Perisse, E., Yin, Y., Lin, A.C., Lin, S., Huetteroth, W., and Waddell, S. (2013). Different kenyon cell populations drive learned approach and avoidance in Drosophila. Neuron *79*, 945–956.

Pitman, J.L., Huetteroth, W., Burke, C.J., Krashes, M.J., Lai, S.L., Lee, T., and Waddell, S. (2011). A pair of inhibitory neurons are required to sustain labile memory in the Drosophila mushroom body. Curr. Biol. *21*, 855–861.

Plaçais, P.Y., Trannoy, S., Friedrich, A.B., Tanimoto, H., and Preat, T. (2013). Two pairs of mushroom body efferent neurons are required for appetitive long-term memory retrieval in Drosophila. Cell Rep. *5*, 769–780.

Pologruto, T.A., Sabatini, B.L., and Svoboda, K. (2003). ScanImage: flexible software for operating laser scanning microscopes. Biomed. Eng. Online *2*, 13.

Qin, H., Cressy, M., Li, W., Coravos, J.S., Izzi, S.A., and Dubnau, J. (2012). Gamma neurons mediate dopaminergic input during aversive olfactory memory formation in Drosophila. Curr. Biol. *22*, 608–614.

Riemensperger, T., Völler, T., Stock, P., Buchner, E., and Fiala, A. (2005). Punishment prediction by dopaminergic neurons in Drosophila. Curr. Biol. *15*, 1953–1960.

Schwaerzel, M., Heisenberg, M., and Zars, T. (2002). Extinction antagonizes olfactory memory at the subcellular level. Neuron *35*, 951–960.

Schwaerzel, M., Monastirioti, M., Scholz, H., Friggi-Grelin, F., Birman, S., and Heisenberg, M. (2003). Dopamine and octopamine differentiate between aversive and appetitive olfactory memories in Drosophila. J. Neurosci. *23*, 10495–10502.

Séjourné, J., Plaçais, P.Y., Aso, Y., Siwanowicz, I., Trannoy, S., Thoma, V., Tedjakumala, S.R., Rubin, G.M., Tchénio, P., Ito, K., et al. (2011). Mushroom body efferent neurons responsible for aversive olfactory memory retrieval in Drosophila. Nat. Neurosci. *14*, 903–910.

Shang, Y., Claridge-Chang, A., Sjulson, L., Pypaert, M., and Miesenböck, G. (2007). Excitatory local circuits and their implications for olfactory processing in the fly antennal lobe. Cell *128*, 601–612.

Tanaka, N.K., Tanimoto, H., and Ito, K. (2008). Neuronal assemblies of the Drosophila mushroom body. J. Comp. Neurol. *508*, 711–755.

Tempel, B.L., Bonini, N., Dawson, D.R., and Quinn, W.G. (1983). Reward learning in normal and mutant Drosophila. Proc. Natl. Acad. Sci. USA *80*, 1482–1486.

Tully, T., and Quinn, W.G. (1985). Classical conditioning and retention in normal and mutant Drosophila melanogaster. J. Comp. Physiol. A Neuroethol. Sens. Neural Behav. Physiol. *157*, 263–277.

Vosshall, L.B. (2000). Olfaction in Drosophila. Curr. Opin. Neurobiol. *10*, 498–503.

Waddell, S. (2013). Reinforcement signalling in Drosophila; dopamine does it all after all. Curr. Opin. Neurobiol. *23*, 324–329.

Wang, Y., Guo, H.F., Pologruto, T.A., Hannan, F., Hakker, I., Svoboda, K., and Zhong, Y. (2004). Stereotyped odor-evoked activity in the mushroom body of Drosophila revealed by green fluorescent protein-based Ca2+ imaging. J. Neurosci. *24*, 6507–6514.

Wang, Y., Mamiya, A., Chiang, A.S., and Zhong, Y. (2008). Imaging of an early memory trace in the Drosophila mushroom body. J. Neurosci. *28*, 4368–4376.

Wang, K., Gong, J., Wang, Q., Li, H., Cheng, Q., Liu, Y., Zeng, S., and Wang, Z. (2014). Parallel pathways convey olfactory information with opposite polarities in Drosophila. Proc. Natl. Acad. Sci. USA *111*, 3164–3169.

Wilson, R.I. (2013). Early olfactory processing in Drosophila: mechanisms and principles. Annu. Rev. Neurosci. *36*, 217–241.

Wong, A.M., Wang, J.W., and Axel, R. (2002). Spatial representation of the glomerular map in the Drosophila protocerebrum. Cell *109*, 229–241.

Xie, Z., Huang, C., Ci, B., Wang, L., and Zhong, Y. (2013). Requirement of the combination of mushroom body γ lobe and α/β lobes for the retrieval of both aversive and appetitive early memories in Drosophila. Learn. Mem. *20*, 474–481.

Yoshihara, M. (2012). Simultaneous recording of calcium signals from identified neurons and feeding behavior of Drosophila melanogaster. J. Vis. Exp. http://dx.doi.org/10.3791/3625.

Yu, D., Keene, A.C., Srivatsan, A., Waddell, S., and Davis, R.L. (2005). Drosophila DPM neurons form a delayed and branch-specific memory trace after olfactory classical conditioning. Cell *123*, 945–957.

Yu, D., Akalal, D.B., and Davis, R.L. (2006). Drosophila alpha/beta mushroom body neurons form a branch-specific, long-term cellular memory trace after spaced olfactory conditioning. Neuron *52*, 845–855.

Zhang, S., and Roman, G. (2013). Presynaptic inhibition of gamma lobe neurons is required for olfactory learning in Drosophila. Curr. Biol. *23*, 2519–2527.

Neuron
Supplemental Information

Activity of Defined Mushroom Body Output Neurons Underlies Learned Olfactory Behavior in *Drosophila*

David Owald, Johannes Felsenberg, Clifford B. Talbot, Gaurav Das, Emmanuel Perisse, Wolf Huetteroth, and Scott Waddell

Inventory for Supplemental Information

Figure S1. (A) Additional data for GRASP experiment in Figure 1F. Whole brain expression pattern of R48B04-LexA driver line used for GRASP in Figure 1F. (B) Similar GRASP signal observed with independent dopaminergic and output neuron driver lines; R58E02-LexA and R66C08-GAL4.

Figure S2. Permissive temperature controls for experiments in Figure 2.

Figure S3. Odor-evoked GCaMP responses in the gamma lobe output to complement responses shown in Figure 3A-D.

Figure S4. Physiological responses to additional odors and behavioral memory tests using these odors. (A) relates to Figure 3A-D. (B) relates to Figure 2A and Figure 3.

Figure S5. Permissive temperature controls for experiments in Figure 3E-F.

Figure S6. Normalized raw data traces for Figure 3I-L.

Figure S7. Example data analysis for odor response curves shown in Figure 3I-J.

Figure S8. Permissive temperature controls for experiments in Figure 4.

Movie S1. Projection view of innervation relating to Figure 1A.

Movie S2. Projection view of innervation relating to Figure 1B.

Movie S3. Projection view of innervation relating to Figure 1C.

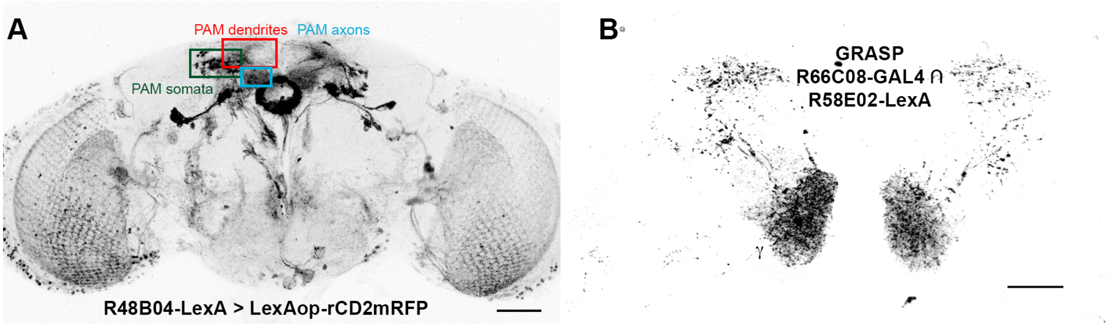

Figure S1. (A) Projection view of a brain from a R48B04-LexA; LexAop-rCD2-mRFP fly. Note that the ellipsoid body expression is in a different plane to that of PAM cells. Scale bar is 50 μm. (B) GRASP reveals sites of putative contact between M6 neurons and the rewarding dopaminergic neurons. Dopaminergic neurons expressed one half of the split GFP with R58E02-LexA and the complementary fragment was driven in M6/MBON-γ5β´2a neurons by R66C08-GAL4. Also compare Figure 1F for GRASP with dopaminergic neurons labeled with R48B04-LexA and M6/MBON-γ5β´2a and M4β´/MBON-β´2mp with VT1211-GAL4. Scale bar is 20 μm.

	immediate appetitive	24h appetitive	immediate aversive
R66C08-GAL4	n = 10<br>0.34 ± 0.04<br>p > 0.05	n = 7<br>0.40 ± 0.04<br>p > 0.05	n = 15<br>-0.55 ± 0.04<br>p > 0.05 ♯
UAS-*shi*^{ts1}	n = 11<br>0.24 ± 0.06<br>p > 0.05	n = 5<br>0.47 ± 0.04 ‡<br>p > 0.05	n = 6<br>-0.34 ± 0.04<br>p > 0.05 ♯
R66C08 > *shi*^{ts1}	n = 10<br>0.28 ± 0.05<br>p > 0.05	n = 4<br>0.50 ± 0.04<br>p > 0.05	n = 12<br>-0.45 ± 0.04<br>p > 0.05
VT1211-GAL4	n = 9<br>0.21 ± 0.02<br>p > 0.05	n = 5<br>0.32 ± 0.04<br>p > 0.05	n = 10<br>-0.36 ± 0.04<br>p > 0.05
UAS-*shi*^{ts1}	n = 9<br>0.30 ± 0.04<br>p > 0.05	n = 5<br>0.47 ± 0.04 ‡<br>p > 0.05	n = 10<br>-0.31 ± 0.07<br>p > 0.05
VT1211 > *shi*^{ts1}	n = 9<br>0.21 ± 0.03<br>p > 0.05	n = 7<br>0.29 ± 0.07<br>p > 0.05	n = 11<br>-0.29 ± 0.05<br>p > 0.05
R21D02-GAL4	n = 8<br>0.30 ± 0.06<br>p > 0.05	n = 6<br>0.16 ± 0.04<br>p > 0.05	n = 4<br>-0.23 ± 0.08<br>p > 0.05
UAS-*shi*^{ts1}	n = 6<br>0.27 ± 0.01<br>p > 0.05	n = 7<br>0.20 ± 0.03<br>p > 0.05	n = 4<br>-0.17 ± 0.04<br>p > 0.05
R21D02 > *shi*^{ts1}	n = 7<br>0.25 ± 0.07<br>p > 0.05	n = 7<br>0.10 ± 0.03<br>p > 0.05	n = 4<br>-0.17 ± 0.04<br>p > 0.05

Figure S2. Permissive temperature control experiments for the restrictive temperature memory experiments shown in Figure 2. ‡ indicates data sets acquired in parallel. # indicates the R66C08-GAL4 and UAS-*shi*^{ts1} flies were statistically different. Data shown are mean ± SEM.

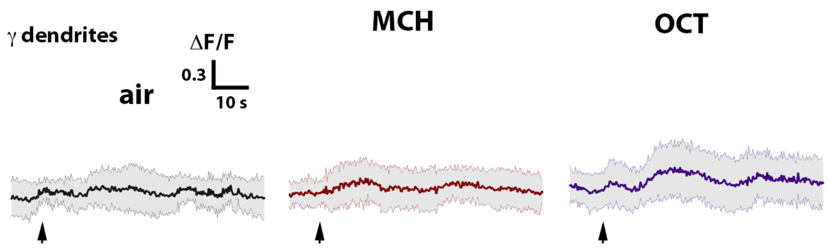

Figure S3. Time courses of odor-evoked GCaMP responses (ΔF/F) collected at the level of the M6/MBON-γ5β´2a neuron dendrites in the γ lobe. Traces represent the mean odor responses (solid line) and standard deviation (grey shading), *n* = 19. Arrows indicate onset of odor presentation. Also compare Figure 3C for M4β´/MBON-β´2mp responses.

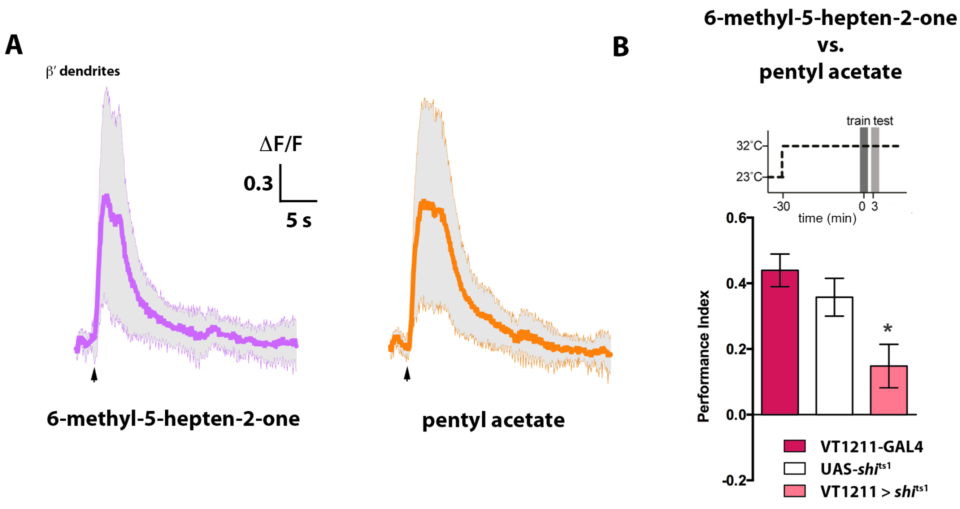

Figure S4. (A) Time courses of odor-evoked GCaMP responses (ΔF/F) collected from M4β′/MBON-β′2mp dendrites to a 5 s 6-methyl-5-hepten-2-one and pentyl acetate exposure. Traces represent the mean odor responses (solid line) and standard deviation (grey shading), n = 16. Arrows indicate onset of odor presentation. (B) Blocking M4β′/MBON-β′2mp and M6/MBON-γ5β′2a (VT1211-Gal4) neurons with UAS-*shibire*^{ts1} significantly impairs 3 min appetitive memory performance when trained with 6-methyl-5-hepten-2-one and pentyl acetate ($n \geq 8$, $P < 0.05$). Data shown are mean ± SEM.

	2 h appetitive	2 h aversive
**VT1211-GAL4**	n = 5<br>0.14 ± 0.07<br>p > 0.05	n = 12<br>-0.18 ± 0.05<br>p > 0.05
**UAS-*shi*^{ts1}**	n = 6<br>0.21 ± 0.05<br>p > 0.05	n = 12<br>-0.16 ± 0.03<br>p > 0.05
**VT1211 > *shi*^{ts1}**	n = 5<br>0.16 ± 0.07<br>p > 0.05	n = 13<br>-0.19 ± 0.05<br>p > 0.05

Figure S5. Permissive temperature controls for the behavioral experiments shown in Figure 3. Data shown are mean ± SEM.

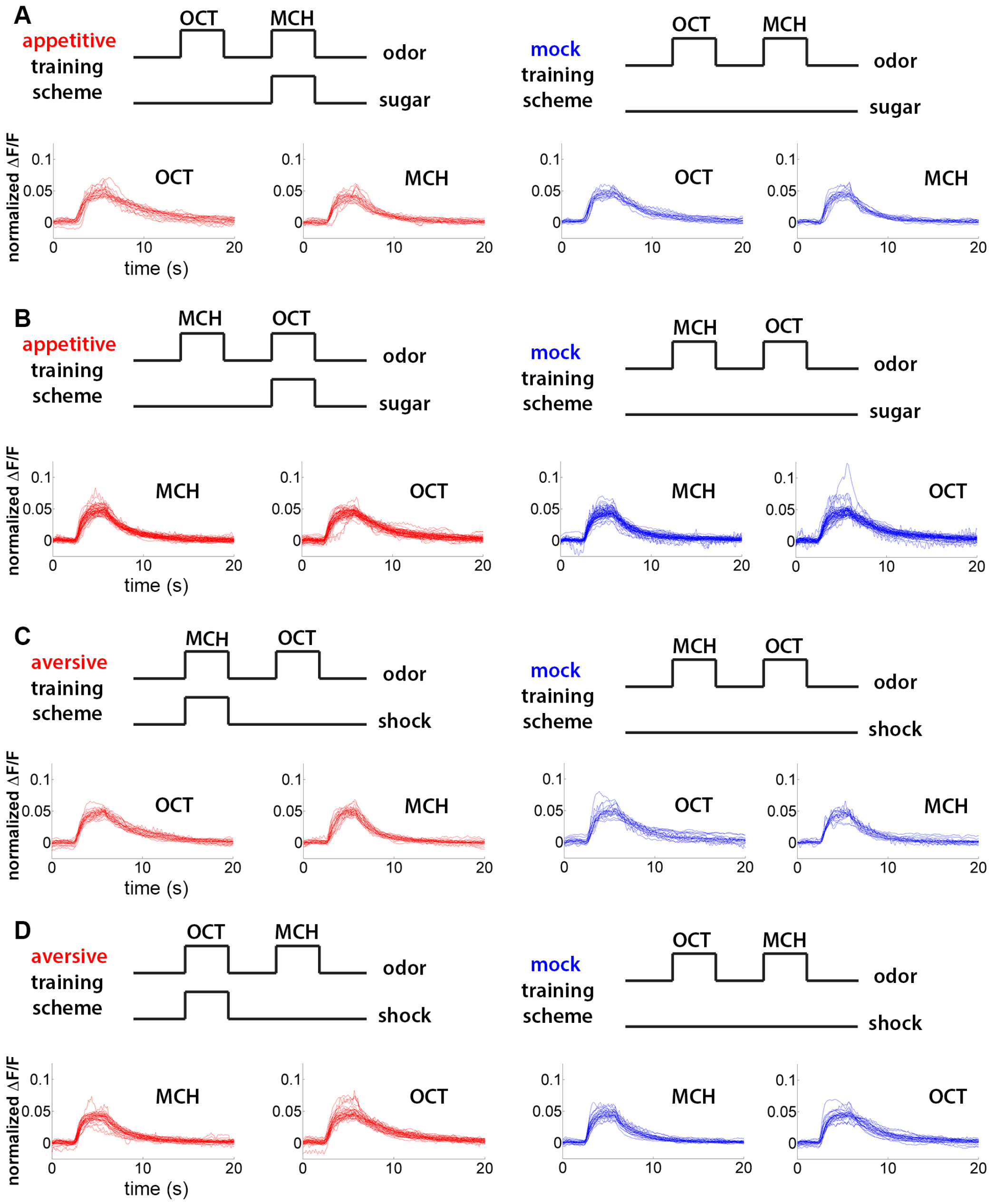

Figure S6. Response traces for each 'n' for data shown in Figure 3I-L. Individual traces are averages of two odor presentations as described in the methods. Conditioning protocols are illustrated in upper panels of A to D and normalized response traces (see methods, equation (i)) are shown in lower panels. (A) appetitive, MCH is CS+ (B) appetitive, OCT is CS+ (C) aversive, MCH is CS+ (D) aversive, OCT is CS+.

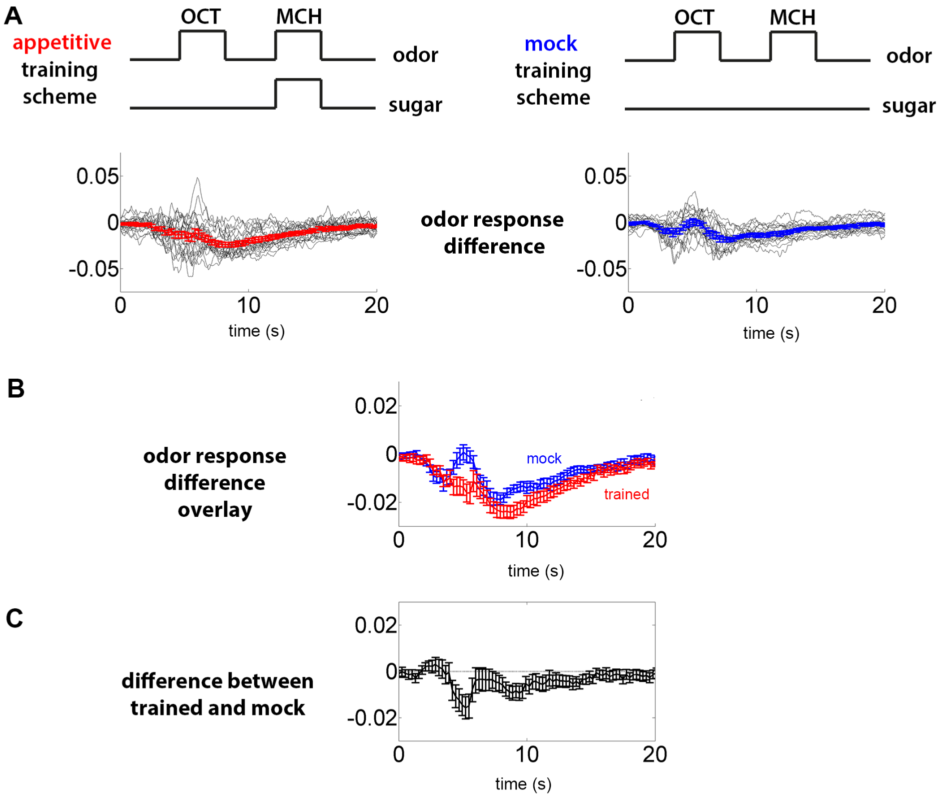

Figure S7. Example of data analysis for odor response curves shown in Figure 3I and 3J. (A) Above: Schematic of the conditioning protocol. Below: The odor response difference curves (see methods). OCT traces are subtracted from the MCH traces per individual trained fly (grey lines). The red (trained) and blue (mock trained) lines are both mean ± SEM. (B) Overlay of the averaged odor response difference curves for the trained (red line ± SEM) and mock trained (blue line ± SEM) flies. (C) The mean odor response difference curves are subtracted in order to compare the averaged responses from trained flies to those from mock trained flies, mean ± SEM. Methods contain further detail.

	MCH	OCT
R21D02-Gal4	n = 13<br>-0.33 ± 0.07<br>$p > 0.05$	n = 10<br>-0.16 ± 0.07<br>$p > 0.05$
VT1211-Gal4	n = 13<br>-0.33 ± 0.05<br>$p > 0.05$	n = 7<br>-0.32 ± 0.05<br>$p > 0.05$
R66C08-Gal4	see below	n = 6<br>-0.48 ± 0.08<br>$p > 0.05$
UAS-*shi*^{ts1}	n = 15<br>-0.62 ± 0.03 #<br>$p < 0.05$	n = 10<br>-0.32 ± 0.07<br>$p > 0.05$
R21D02 > *shi*^{ts1}	n = 10<br>-0.21 ± 0.06<br>$p > 0.05$	n = 7<br>-0.33 ± 0.07<br>$p > 0.05$
VT1211 > *shi*^{ts1}	n = 15<br>-0.35 ± 0.04<br>$p > 0.05$	n = 6<br>-0.36 ± 0.13<br>$p > 0.05$
R66C08 > *shi*^{ts1}	see below	n = 6<br>-0.45 ± 0.09<br>$p > 0.05$
R66C08-Gal4	n = 6<br>-0.38 ± 0.03<br>$p < 0.05$	see above
UAS-*shi*^{ts1}	n = 6<br>-0.23 ± 0.09<br>$p < 0.05$	see above
R66C08 > *shi*^{ts1}	n = 6<br>-0.30 ± 0.09<br>$p > 0.05$	see above

Figure S8. Permissive temperature control experiments for the restrictive naïve odor choice experiments shown in Figure 4. # indicates the UAS-*shi*^{ts1} flies showed statistical difference to relevant groups. Data shown are mean ± SEM.
